# Quantum higher-order Fourier analysis and the Clifford hierarchy

**DOI:** 10.1073/pnas.2515667122

**Published:** 2025-11-07

**Authors:** Kaifeng Bu, Weichen Gu, Arthur Jaffe

**Affiliations:** ^a^Department of Mathematics, The Ohio State University, Columbus, OH 43210; ^b^Department of Physics, Harvard University, Cambridge, MA 02138; ^c^Department of Statistics, University of New Hampshire, Durham, NH 03824; ^d^Departments of Mathematics, Harvard University, Cambridge, MA 02138

**Keywords:** Fourier analysis, quantum, Clifford hierarchy, higher order

## Abstract

We propose a mathematical framework that we call quantum, higher-order Fourier analysis. This generalizes the classical theory of higher-order Fourier analysis, which led to many recent advances in number theory and combinatorics. We define a family of “quantum measures” on linear transformations on a Hilbert space, that reduce in the case of diagonal matrices to the uniformity norms introduced by Timothy Gowers. We show that our quantum measures and our related theory of quantum higher-order Fourier analysis characterize the Clifford hierarchy, an important notion of complexity in quantum computation. In particular, we give a necessary and sufficient analytic condition that a unitary is an element of the kth level of the Clifford hierarchy.

## Significance

Quantum Fourier analysis is a powerful tool in mathematical physics. Here, we introduce quantum, higher-order Fourier analysis (q-HOFA). We explore some mathematical properties of this theory and show that it provides a way to quantify the complexity of unitary gates in quantum computation. This should provide a natural starting point for a more general study of q-HOFA.

This paper revolves about two interrelated themes. First, we present the foundation for a mathematical theory that we call quantum, higher-order Fourier analysis (q-HOFA). We introduce a family of quantum uniformity measures ${\left\|\cdot \right\|}_{Q^{k}}$ on linear transformations B, given explicitly in Eq. **7**. We show that ${\left\|B\right\|}_{Q^{k}}$ is nonnegative and increases monotonically in k. We prove that these measures are norms for k=2,3. These properties of the measures are sufficient to establish the resulting applications to quantum information that we consider here; the norm property has not proved necessary.

These measures generalize the classical uniformity norms introduced by Gowers (1), which led to the theory of classical, higher-order Fourier analysis. The classical theory has been studied widely and provides many useful insights into classical combinatorics and number theory. The present paper provides an analysis of the corresponding quantum theory.

Our second theme is to show that q-HOFA gives an analytical measure of the complexity of quantum gates. A widely used algebraic classification of the complexity of a unitary gate in quantum information theory is the Clifford hierarchy, introduced by Gottesman and Chuang (2). This hierarchy has been extensively studied. We show here that our quantum uniformity measures quantify the Clifford hierarchy analytically. Among other things, we prove that a unitary U belongs to the kth-level of the Clifford hierarchy, if and only if ${\left\|U\right\|}_{Q^{k+1}}=1$. We believe that the analytic notions of q-HOFA will find other applications in both mathematics and in physics.

## 1.1. Background.

In classical Fourier analysis one pairs the function f(x) with the linear exponential $e^{\mathrm{ipx}}$. One obtains higher-order, classical Fourier analysis by generalizing this procedure to pair f(x) with the exponential $e^{iP_{k}\left(x\right)}$ of a polynomial $P_{k}\left(x\right)$ of degree k (1, 3–5). Here $P_{k}\left(x\right)$ is called a phase polynomial.

One can characterize the degree k of $P_{k}$ inductively, by using a discrete, nonlinear, multiplicative derivative $\partial_{a}$. Suppose that a function $f\left(x\right)=e^{ig(x)}$ that takes values on the unit circle in the complex plane. In classical analysis, one defines the discrete multiplicative derivative as $\partial_{a}f\left(x\right):=f\left(x+a\right)\bar{f(x)}=e^{i\left(g(x+a)-g(a)\right)}$. In case that $g\left(x\right)=P_{k+1}\left(x\right)$ is a polynomial of degree k+1, its derivative is a polynomial of degree k.

Gowers quantified pairing with a phase polynomial by introducing a family of uniformity norms (1), that provide a remarkable, quantitative bound for Szemerédi’s theorem on arithmetic progressions (3). These classical norms ${\left\|\cdot \right\|}_{U^{k}}$ have the form

[1] $$\begin{array}{c} {\left\|f\right\|}_{U^{k}}^{2^{k}}=E_{{\vec{a}}_{1},...,{\vec{a}}_{k},\vec{x}}\left(\partial_{{\vec{a}}_{1},..,{\vec{a}}_{k}}f\left(\vec{x}\right)\right)\;, \end{array}$$

where the expectation E is a uniform average over the ${\vec{a}}_{j}$ and $\vec{x}$, where ${\vec{a}}_{j},\vec{x}\in F_{2}^{n}$ and f is function defined on $F_{2}^{n}$, namely n copies of the finite field with two elements.

The further development of that work led to other applications in number theory, in additive combinatorics, and in theoretical computer science. Many mathematicians including Gowers, Green, Tao, Szegedy, Host, B. Kra, and Ziegler, made significant contributions to build a comprehensive theory of higher-order Fourier analysis; see, e.g. refs. 3, and 6–13. One has used the classical Gowers 3-norm to study vector states (14–17).

Here we extend the ideas in classical, higher-order Fourier analysis to the quantum case. We study the configuration space of quantum theory as a noncommutative Weyl group. We define quantum uniformity measures on transformations on this space in Eq. **7** and study the properties of these measures. Discrete classical analysis corresponds to restricting the Weyl group to a certain commutative subgroup, in which case our quantum measures reduce to Gowers’ classical uniformity norms. After studying properties of the quantum uniformity measures, we show their relevance to complexity theory in quantum computation.

## 1.2. Quantum Derivative.

In this paper, we replace the underlying space $F_{2}$ by a noncommutative (quantum) phase space, equipped with a corresponding family of noncommutative translations. In continuum quantum physics, the Weyl group describes such a phase space, and it has been widely studied. In the next section we introduce a discrete quantum version of this framework that is the basis for much work in quantum information theory. In a different context, Gross (18) (and others) have extensively studied this discrete group as a quantum phase space.

We use unitary translations on the quantum phase space to define a discrete quantum derivative. Using this derivative, we define polynomials of degree k inductively. This leads one to introduce quantum higher-order Fourier analysis, which involves the pairing of a unitary U with the exponential of a polynomial of degree k. This set of ideas provides the path to quantum, higher-order Fourier analysis.

This mathematical framework works across different settings where translations define a discrete directional derivative. Define the translation of a unitary B in the direction given by a unitary A as the conjugation $ABA^{*}$. One then obtains the discrete, multiplicative, directional derivative $\partial_{A}B$ of B as

[2] $$\begin{array}{c} \partial_{A}B=\left(ABA^{*}\right)B^{*}=:{\left[A,B\right]}_{c}\;. \end{array}$$

Throughout this paper we denote the commutator ${\left[\;,\;\right]}_{c}$ by the expression Eq. **2**, which agrees with the group commutator in case A,B are both unitaries.

## 1.3. Weyl Operators and Clifford Unitaries.

Unitary matrices X and Z play a central role in quantum theory. One denotes the eigenbasis (or computational basis) for Z by |k〉, where $k\in Z_{d}$, and defines $Z|k\rangle =\omega^{k}|k\rangle$. Here $\omega =e^{\frac{2\pi i}{d}}$. Also X|k〉=|k+1〉, interpreted modulo d. These transformations do not commute, but satisfy the relations,

$${\left[X^{q},Z^{p}\right]}_{c}=X^{q}Z^{p}X^{-q}Z^{-p}=\omega^{-qp}\;.$$

For $a=\left(p,q\right)\in V=Z_{d}^{2}$, and for $\zeta =e^{\frac{\pi i(d+1)}{d}}$ a square root of ω, define the unitary Weyl operator w(a) as

[3] $$\begin{array}{c} w\left(a\right)=\zeta^{-pq}Z^{p}X^{q}, \end{array}$$

These unitaries generate the 1-qudit Weyl group, known as the Pauli group in the quantum literature. Taking an n-fold tensor product yields the n-qudit Weyl group.

The Weyl operators are an orthonormal Fourier basis with respect to the inner product

[4] $$\begin{array}{c} \langle A,B\rangle =\frac{1}{d^{n}}\mathrm{Tr}\left\{A^{*}B\right\}\;,\;\;\text{so}\;A=\sum_{\vec{a}\in Z_{d}^{2n}}\Xi_{A}\left(\vec{a}\right)\;w\left(\vec{a}\right)\;. \end{array}$$

Conjugation by the unitary $A=w(\vec{a})$ defines translation on quantum phase space by $\vec{a}$,

[5] $$\begin{array}{c} B\mapsto B\left(\vec{a}\right)=w\left(\vec{a}\right)\;B\;w{\left(\vec{a}\right)}^{*}\;. \end{array}$$

## 1.4. Stabilizers and Classical Computation.

There is an important family of quantum states introduced by Gottesman (19) and defined as common eigenstates of a commuting subgroup of Weyl operators. They are called “stabilizer states;” see *Definition* 13. The elegant mathematical structure of stabilizer states allows them to be precisely described and manipulated. This makes them invaluable in the development of quantum error correction, where they form the basis of stabilizer codes, a class of codes capable of protecting quantum information from errors. Notable examples of stabilizer codes include Shor’s 9-qubit code (20) and Kitaev’s toric code (21). Along with the stabilizer states, there is an important family of unitaries called Clifford unitaries, which map Weyl operators by conjugation to Weyl operators.

The famous Gottesman–Knill theorem is the statement that quantum circuits with stabilizer states as input, Clifford unitaries as evolution, and measurement in the computational basis, can be efficiently simulated by a classical computer (22). This result delineates a boundary between classical and quantum computational power, and emphasizes the importance of non-Clifford gates in achieving quantum computational advantage. Many other results on classical simulation appear in the literature (14, 23–30). Stabilizer states are the quantum equivalent of classical Gaussians (31–33).

One way to quantify the complexity of non-Clifford gates is the Clifford hierarchy, introduced by Gottesman and Chuang (2). This classification helps to implement fault-tolerant quantum computation (2). In this work, we explore these ideas through the lens of q-HOFA.

## 1.5. Quantum Uniformity Measures.

Using the translation implemented by the Weyl operators, we define the phase-space derivative,

[6] ∂a→B:=∂w(a→)B=B(a→)B∗,

as specified in Eqs. **2** and **5**. Multiple derivatives are defined iteratively as $\partial_{{\vec{a}}_{k},{\vec{a}}_{k-1},\ldots ,{\vec{a}}_{1}}=\partial_{{\vec{a}}_{k}}\partial_{{\vec{a}}_{k-1},\ldots ,{\vec{a}}_{1}}$. The quantum uniformity measures are bounded by the operator norm:

[7] ‖B‖Qk2k=Ea→k,...,a→1∈Vn1dnTr∂a→k,...,a→1B⩽‖B‖2k.

The expectation E denotes an average over each ${\vec{a}}_{j}\in Z_{d}^{2n}$. We develop properties of these measures in §5. In §10, we show that these measures can also be characterized by the quantum convolution proposed earlier by the authors in refs. 31, 32, 34, and 35.

We compare some properties of the classical and quantum Fourier analysis in Table 1. This theory gives rise to quantum phase polynomials of degree-k, generalizing classical phase polynomials that occur in classical combinatorics. The quantum uniformity norms and the Clifford hierarchy, on which we elaborate here, provide a natural starting point for a more general study of q-HOFA.

**Table 1.** A comparison of q-HOFA with c-HOFA

	Quantum, higher-order Fourier analysis	Classical, higher-order Fourier analysis
Derivative	$\partial_{A}B=\left(ABA^{*}\right)B^{*}$	$\partial_{\vec{w}}f\left(\vec{x}\right)=f\left(\vec{x}+\vec{w}\right)\bar{f(\vec{x})}$
Degree-1 polynomial	Weyl operator $w(\vec{a})$	Phase linear function ${\left(-1\right)}^{\vec{a}\cdot \vec{x}}$
Fourier coefficient	$\Xi_{U}\left[\vec{a}\right]=\left\langle U,w\left(\vec{a}\right)\right\rangle$	$\hat{f}\left(\vec{a}\right)=\left\langle f,{\left(-1\right)}^{\vec{a}\cdot x}\right\rangle$
Degree-k polynomial	kth level Clifford hierarchy $C^{\left(k\right)}$	Phase polynomial ${\left(-1\right)}^{g(\vec{x})}$, deg(g)⩽k, denote the set as $D_{k}$
Higher-order Fourier coefficient	$\left\langle U,V\right\rangle ,\forall V\in C^{\left(k\right)}$	⟨f,g⟩, $\forall g\in D_{k}$
Uniformity measures	Quantum uniformity measure ${\left\|\cdot \right\|}_{Q^{k}}$ (*Definition* 25)	Gowers norm ${\left\|\cdot \right\|}_{U^{k}}$ Eq. **1**
Monotonicity	${\left\|\cdot \right\|}_{Q^{k}}⩽{\left\|\cdot \right\|}_{Q^{k+1}}$ (*Theorem* 38)	${\left\|\cdot \right\|}_{U^{k}}⩽{\left\|\cdot \right\|}_{U^{k+1}}$
Connection with $L^{p}$ norm	${\left\|\cdot \right\|}_{Q^{k}}⩽{\left\|\cdot \right\|}_{p_{k}}$ with $p_{k}=\frac{2^{k}}{k+1}$ (*Theorem* 46)	$\left\|\cdot \right\|x_{U^{k}}⩽{\left\|\cdot \right\|}_{p_{k}}$ with $p_{k}=\frac{2^{k}}{k+1}$
Log-convexity	${\left\|\rho \right\|}_{Q^{2}}⩽{\left\|\rho \right\|}_{Q^{1}}^{1/2}{\left\|\rho \right\|}_{Q^{3}}^{1/2}$, for pure state ρ (*Theorem* 49)	${\left\|f\right\|}_{U^{2}}⩽{\left\|f\right\|}_{U^{1}}^{1/2}{\left\|f\right\|}_{U^{3}}^{1/2}$ for positive function f
Application in property testing	Clifford-hierarchy testing (*Theorem* 57)	Low-degree polynomial testing

## 1.6. Summary of Main Results.

Theorem 1 (Positivity, Monotonocity, and Norm). *For any* n*-qudit linear operator* B,

$$0⩽{\left\|B\right\|}_{Q^{k}}⩽{\left\|B\right\|}_{Q^{k+1}},\;\;\text{for}\;1⩽k\;.$$

*Furthermore,* ${\left\|\cdot \right\|}_{Q^{k}}$ *is a norm for* k=2,3.

In *Propositions* 26 and 27 and §9 we prove this and other relations for the quantum uniformity measures, including the two following propositions:

Proposition 2. *For arbitrary* B,

$$\begin{array}{ll} \|B{\|}_{Q^{1}} & =|\frac{1}{d^{n}}\text{Tr}\left\{B\right\}|\;,\;\|B{\|}_{Q^{2}}=\|{\text{Ξ}}_{B}{\|}_{l^{4}},\\ \|B{\|}_{Q^{k}}^{2^{k}} & =E_{\vec{a}}\|\partial_{\vec{a}}B{\|}_{Q^{k-1}}^{2^{k-1}}\\ & =\underset{{\vec{a}}_{1},...,{\vec{a}}_{k-1}\in V^{n}}{E}|\frac{1}{d^{n}}\text{Tr}\left\{\partial_{{\vec{a}}_{k-1},...,{\vec{a}}_{1}}B\right\}{|}^{2}\;,k⩾2. \end{array}$$

Corollary 3. *If* $\partial_{{\vec{a}}_{k-1},\ldots ,{\vec{a}}_{1}}B=\lambda^{2^{k-1}}I$ *is a scalar multiple of the identity, then* ${\left\|B\right\|}_{Q^{k^{\prime }}}=\lambda$, *for* $k^{\prime }⩾k$.

Proposition 4. *The* $Q^{k}$ *measure of a pure state* $\rho =\sum_{\vec{a}\in V^{n}}\Xi_{\rho }\left(\vec{a}\right)\;w\left(\vec{a}\right)$ *is given by* $\ell^{p}$ *norms of its Fourier coefficients* $\Xi_{\rho }\left(\vec{a}\right)$. *See Proposition 47 for the exact statement*.

The Clifford hierarchy $C^{\left(k\right)}$, see *Definition* 16, is an important algebraic concept used to characterize the complexity of unitaries in quantum computation. Our quantum uniformity measures give an analytic characterization of the Clifford hierarchy.

Theorem 5 (Analytic Characterization of $C^{\left(k\right)}$). *A unitary* $U\in C^{\left(k\right)}$, *iff* ${\left\|U\right\|}_{Q^{k+1}}=1$.

We complete the proof of Theorem 5 in *Proposition* 54. While $\|U{\|}_{Q^{1}}=\|U^{*}{\|}_{Q^{1}}$, in general for k>1, the unitaries U and $U^{*}$ have different quantum uniformity norms ${\left\|\cdot \right\|}_{Q^{k}}$. In *Corollary* 24, we give a class of unitaries $U\in C^{\left(k\right)}$ which are closed under taking their adjoint.

Theorem 6 (The Classical Case). *Let* U *be diagonal in the computational basis, with diagonal values* f. *Then* ${\left\|U\right\|}_{Q^{k}}={\left\|f\right\|}_{U^{k}}$, *the Gowers’ uniformity norm*.

Next we address a different aspect of quantum Fourier analysis, namely the magnitude of the overlap between a unitary and $C^{\left(k\right)}$.

Definition 7 (Overlap with the Hierarchy): The hierarchy-overlap measure of a unitary U with the k-th level of the Clifford hierarchy is

$$\begin{array}{c} {\left\|U\right\|}_{q^{k+1}}=\;\max_{V\in C^{\left(k\right)}}{\left|\left\langle V,U\right\rangle \right|}^{2}. \end{array}$$

We have the following relation between that hierarchy-overlap measure and the quantum uniformity norms:

Theorem 8 (Direct Inequality). *Given an* n*-qudit unitary* U,

$$\begin{array}{c} \begin{array}{c} \|U{\|}_{q^{k}}⩽\|U{\|}_{Q^{k}},\;\;for\;1⩽k\;. \end{array} \end{array}$$

*Furthermore* $\|U{\|}_{Q^{k}}=1$, *iff* $\|U{\|}_{Q^{k}}=1$. *Thus* $U\in C^{\left(k\right)}$, *iff* ${\left\|U\right\|}_{q^{k+1}}=1$.

Conjecture 9 (Inverse Inequality Conjecture). *If* $0<c⩽{\left\|U\right\|}_{Q^{k}}$, *does there exist* d, *independent of* n, *such that* $0<d⩽{\left\|U\right\|}_{q^{k}}$*?*

## 2. Preliminaries

An n-qudit system is based on a Hilbert space $H^{⊗n}$, where $H≃C^{d}$. The chosen natural number d characterizes the system and we take d to be prime. Let $L(H^{⊗n})$ denote the set of all linear transformations on $H^{⊗n}$, and let $D(H^{⊗n})$ denote the set of all quantum states on $H^{⊗n}$.

Let a=(p,q)∈V denote a point in the phase space V. For d an odd prime, the one-qudit Weyl operators Eq. **3** is

[8] $$\begin{array}{c} w\left(a\right)=\omega^{-2^{-1}pq}Z^{p}X^{q}\;, \end{array}$$

where $2^{-1}=\frac{d+1}{2}$ denotes the inverse of 2 in $Z_{d}$. If d=2, take the Weyl operators to be

[9] $$\begin{array}{c} w\left(a\right)=i^{-pq}Z^{p}X^{q}\;. \end{array}$$

The Weyl operators satisfy $w{\left(a\right)}^{*}=w\left(-a\right)$, and their multiplication is given by a symplectic product ${\left\langle a,a^{\prime }\right\rangle }_{s}$ on phase space, ${\langle a,a^{\prime }\rangle }_{s}=pq^{\prime }-qp^{\prime }$. Then

[10] $$\begin{array}{cc} w\left(a\right)\;w\left(b\right)w{\left(a\right)}^{*} & =\omega^{{\left\langle a,b\right\rangle }_{s}}\;w\left(b\right)\;, \end{array}$$

[11] $$\begin{array}{cc} T\left(a\right):=w\left(a\right)Tw{\left(a\right)}^{*} & =\sum_{b}\omega^{{\left\langle a,b\right\rangle }_{s}}\;\Xi_{T}\left(b\right)w\left(b\right)\;, \end{array}$$

[12] $$\begin{array}{cc} \left(T(a))(b\right) & =T(a+b)\;. \end{array}$$

In the case of n qudits, denote $V^{n}=Z_{d}^{n}\times Z_{d}^{n}\;$. For $\vec{a}\in V^{n}$. Define the Weyl operators $w(\vec{a})$ as tensor products,

$$\begin{array}{c} w\left(\vec{a}\right)=w\left(a_{1}\right)⊗...⊗w\left(a_{n}\right)\;. \end{array}$$

As stated in the introduction,

Proposition 10 (Weyl Orthogonality). *The Weyl operators* ${w(\vec{a})}_{\vec{a}\in V^{n}}$ *form an orthonormal basis in* $L(H^{⊗n})$ *with respect to the inner product* Eq. **4**.

The generators $w(\vec{a})$ of the Weyl group are our quantum Fourier basis. Any linear operator T on $H^{⊗n}$ has a Fourier expansion

[13] $$\begin{array}{c} T=\sum_{\vec{a}}\Xi_{T}\left(\vec{a}\right)w\left(\vec{a}\right)\;,\;\;\text{where}\;\Xi_{T}\left(\vec{a}\right)=\langle w(\vec{a}),T\rangle \;. \end{array}$$

The coefficients $\Xi_{T}\left(\vec{a}\right)$ are called the characteristic function of the n-qudit transformation T on $H^{⊗n}$. This Fourier expansion has extensive uses in a myriad of applications, including quantum boolean functions (36), classical simulation of quantum circuits (14), the quantum circuit complexity (37), nonlocal games based on noisy entangled states (38, 39), the sample complexity of quantum machine learning (40, 41) and many other ways. (See also a more general framework of quantum Fourier analysis in ref. 42).

Definition 11 (Quantum Translation and Expectation): The quantum translation of a transformation T is $T\left(\vec{a}\right)=w\left(\vec{a}\right)\;T\;w{\left(\vec{a}\right)}^{*}$. The expectation $E_{\vec{a}}$ of the $d^{2n}$ possible translations is the unweighted average,

[14] $$\begin{array}{c} E_{\vec{a}}\;T\left(\vec{a}\right)=\frac{1}{d^{2n}}\sum_{\vec{a}\in V^{n}}T\left(\vec{a}\right)\;. \end{array}$$

Proposition 12. *The expectation Eq. **14** over translations equals the normalized trace*,

[15] $$\begin{array}{c} E_{\vec{a}}\;T\left(\vec{a}\right)=\frac{1}{d^{n}}\mathrm{Tr}\left\{T\right\}\;. \end{array}$$

***Proof:*** For $\vec{a}=\left(\vec{p},\vec{q}\right)$ and $\vec{b}=\left({\vec{p}}^{\prime },{\vec{q}}^{\prime }\right)$, the multiplication law Eq. **10** gives

$$\begin{array}{cc} E_{\vec{a}}\;w\left(\vec{a}\right)\;w\left(\vec{b}\right)\;w{\left(\vec{a}\right)}^{*} & =\frac{1}{d^{2n}}\sum_{\vec{a}}\omega^{{\langle \vec{a},\vec{b}\rangle }_{s}}\;w\left(\vec{b}\right)\\ & =\left(\prod_{j=1}^{n}\frac{1}{d^{2}}\sum_{p_{j},q_{j}}\omega^{\left(p_{j}q_{j}^{\prime }-q_{j}p_{j}^{\prime }\right)}\right)\;w\left(\vec{b}\right)=\delta_{\vec{b},\vec{0}}\;. \end{array}$$

Thus $E_{\vec{a}}T\left(\vec{a}\right)=\sum_{\vec{b}\in V}\Xi_{T}\left(\vec{b}\right)\;\delta_{\vec{b},\vec{0}}=\frac{1}{d^{n}}\mathrm{Tr}\left\{T\right\}$.

Definition 13 [Stabilizer state (19, 43)]: A stabilizer vector for an n-qudit system is a vector that is invariant under a maximal abelian subgroup of Weyl operators. The corresponding pure stabilizer state is the projection onto this eigenvector. A general stabilizer state ρ is a convex linear combination of pure stabilizer states.

Stabilizer states encompass a broad class of quantum states, including Bell states and GHZ states, which play a fundamental role in quantum entanglement theory and quantum error correction theory. Several alternative characterizations of stabilizer states are given in refs. 31 and 32.

Proposition 14 (31, 32). *Given an* n*-qudit pure state* ρ=|ψ⟩⟨ψ|, *it is a pure stabilizer state if and only if the absolute value of the characteristic function* $|\Xi_{\rho }\left[\vec{a}\right]|\in \left\{0,\frac{1}{d^{n}}\right\}$, *for any* $\vec{a}\in V^{n}$.

In addition to the stabilizer states, there is another important family of unitaries in quantum information called Clifford unitaries.

Definition 15 (Clifford unitary): An n-qudit unitary U is a Clifford unitary if conjugation by U maps every Weyl operator to another Weyl operator, up to a phase.

It is elementary to see that conjugation by a Clifford unitary maps a stabilizer state to a stabilizer state.

To further understand the computational power of quantum gates beyond Clifford unitaries, the concept of the Clifford hierarchy was introduced by Gottesman and Chuang (2). The hierarchy consists of an increasing family of transformations $C^{\left(k\right)}\subset C^{\left(k+1\right)}$, where $C^{\left(1\right)}$ denotes the group generated by the n-qudit Weyl operators $w(\vec{a})$.

Definition 16 (Clifford hierarchy): For k>1, the kth level $C^{\left(k\right)}$ of the Clifford hierarchy for n qudits is the set of unitaries on $H^{⊗n}$ such that

[16] $$\begin{array}{c} C^{\left(k\right)}:=\left\{U:\;UC^{\left(1\right)}U^{*}\subset C^{\left(k-1\right)}\right\}\;. \end{array}$$

In case that k=2, the set $C^{\left(2\right)}$ is known as the set of all n-qudit Clifford unitaries. In case k=3, the third level of the hierarchy $C^{\left(3\right)}$ includes the T gate and the control–control CCZ gate. These unitaries are used to realize universal quantum computation. Levels of the hierarchy reflect increasing complexity of a quantum gate U.

Aside from unitary transformations, a quantum operation (or channel) is defined as a completely positive and trace-preserving (CPTP) map.

Definition 17 [Choi-Jamiołkowski isomorphism (44, 45)]: Given a quantum channel Λ from $H_{A}$ to $H_{A^{\prime }}$, the Choi state $J_{\Lambda }$ is

$$\begin{array}{c} J_{\Lambda }=id_{A}⊗\Lambda \left(|\Phi \right\rangle \left\langle \Phi |\right)\;, \end{array}$$

where $|\text{Φ}\rangle =\frac{1}{\sqrt{d^{n}}}\sum_{\vec{j}\in {\mathbb{Z}}_{d}^{n}}|\vec{j}\rangle_{A}⊗|\vec{j}\rangle_{A^{\prime }}$ is the maximally entangled state on $H_{A}⊗H_{A^{\prime }}$.

For any input state ρ, the output state of the quantum channel Λ(ρ) can be represented via the Choi state $J_{\Lambda }$ as

[17] $$\begin{array}{c} \Lambda \left(\rho \right)=d^{n}{\mathrm{Tr}}_{A}\left\{J_{\Lambda }\cdot \rho_{A}^{T}⊗I_{A^{\prime }}\right\}\;, \end{array}$$

where $\rho_{A}^{T}$ denotes the transpose of $\rho_{A}$.

## 3. The Quantum Derivative

Given two operators A and B, one can consider the multiplicative, quantum derivative of B in the direction A is Eq. **2**, as $\partial_{A}B=ABA^{*}B^{*}$. Throughout this paper we specialize to the case that $A=w(\vec{a})$ is a Weyl operator, whose conjugation implements a translation in quantum phase space, which we denote as $B\left(\vec{a}\right)=w\left(\vec{a}\right)Bw{\left(\vec{a}\right)}^{*}$. This is a generalization of the notion of the classical discrete derivative, defined by translations on a classical configuration space.

Definition 18 (Quantum Discrete Derivative): The multiplicative quantum derivative of B in the direction $\vec{a}$ is

[18] $$\begin{array}{c} \partial_{\vec{a}}B=\left[w\left(\vec{a}\right),B\right]=w\left(\vec{a}\right)Bw{\left(\vec{a}\right)}^{*}B^{*}=B\left(\vec{a}\right)B^{*}\;. \end{array}$$

The corresponding k-th order derivative is given inductively as

[19] $$\begin{array}{c} \partial_{{\vec{a}}_{k},...,{\vec{a}}_{1}}B=\partial_{{\vec{a}}_{k}}\left(\partial_{{\vec{a}}_{k-1},...,{\vec{a}}_{1}}B\right). \end{array}$$

While the multiplicative quantum derivative $\partial_{\vec{a}}B$ is not linear in B, nor does it satisfy the Leibniz rule. However, the normalized trace satisfies

[20] $$\begin{array}{c} \frac{1}{d^{n}}\mathrm{Tr}\left\{\partial_{\vec{a}}\left(B^{*}C\right)\right\}=\langle \partial_{\vec{a}}B,\partial_{\vec{a}}C\rangle \;. \end{array}$$

Proposition 19 (Expectations of Products and Derivatives). *The expectation defines a nonnegative form, and the expectation of the quantum derivative is nonnegative. In particular,*

[21] $$\begin{array}{c} E_{\vec{a}}\langle C,B(\vec{a})\rangle =\frac{1}{d^{n}}\mathrm{Tr}\left\{B\right\}\;\bar{\frac{1}{d^{n}}\mathrm{Tr}\left\{C\right\}}\;, \end{array}$$

*and*

[22] $$\begin{array}{c} 0⩽E_{\vec{a}}\;\partial_{\vec{a}}B={\left|\frac{1}{d^{n}}\mathrm{Tr}\left\{B\right\}\right|}^{2}\;. \end{array}$$

***Proof:*** The first identity is a consequence of the definition Eqs. **4** and **15**. The second equality follows from setting B=C and using *Definition* 18 of the quantum derivative.

### 3.1 Reduction to the Classical Case.

The quantum derivative reduces to the classical discrete derivative for operators that are diagonal in the computational basis $|\vec{x}\rangle$. Let $f:Z_{d}^{n}\to C$ be a function defining the diagonal operator

[23] $$\begin{array}{c} B_{f}=\sum_{\vec{x}\in Z_{d}^{n}}f\left(\vec{x}\right)|\vec{x}\rangle \langle \vec{x}|\;. \end{array}$$

The multiplicative, classical derivative in direction $\vec{q}$ of a complex-valued function $f(\vec{x})$ on $Z_{d}^{n}$ is

[24] $$\begin{array}{c} \left(\partial_{\vec{q}}f\right)\left(\vec{x}\right)=f\left(\vec{x}+\vec{q}\right)\bar{f(\vec{x})}\;. \end{array}$$

If f takes values on the unit circle, this is a natural definition of a discrete derivative in direction $\vec{q}$ of the phase of f. For f taking arbitrary complex values, this definition is still useful, and one also uses the name “derivative.”

Proposition 20. *Let* $B_{f}$ *have the form Eq. **23**, and let* $\vec{a}=\left(\vec{p},\vec{q}\right)$. *The quantum derivative* $\partial_{\vec{a}}B_{f}$ *is*

[25] $$\begin{array}{c} \partial_{\vec{a}}B_{f}=B_{\partial_{-\vec{q}}f}\;. \end{array}$$

***Proof:*** By definition, the phase in $\omega (\vec{a})$ cancels and

$$\begin{array}{cc} \partial_{\vec{a}}B_{f} & =Z^{\vec{p}}X^{\vec{q}}B_{f}X^{-\vec{q}}Z^{-\vec{p}}{\left(B_{f}\right)}^{*}\\ & =\sum_{\vec{x},\vec{y}}f(\vec{x})\bar{f(\vec{y})}Z^{\vec{p}}|\vec{x}+\vec{q}\rangle \langle \vec{x}+\vec{q}|Z^{-\vec{p}}|\vec{y}\rangle \langle \vec{y}|\\ & =\sum_{\vec{x},\vec{y}}f(\vec{x}-\vec{q})\bar{f(\vec{y})}|\vec{x}\rangle \langle \vec{x}|\vec{y}\rangle \langle \vec{y}|\\ & =\sum_{\vec{x}}f(\vec{x}-\vec{q})\bar{f(\vec{x})}|\vec{x}\rangle \langle \vec{x}|=B_{\partial_{-\vec{q}}f}. \end{array}$$

In the last equality we use the definition Eq. **24** to obtain the claimed result.

## 4. Quantum Polynomials and the Clifford Hierarchy

We use the quantum derivative to give an inductive definition of the exponential $Q^{\left(k\right)}$ of a quantum polynomial of degree k.

Definition 21 (Quantum Polynomials): Let the exponential $Q^{\left(0\right)}$ of a quantum polynomial of degree zero be a scalar multiple of the identity, $Q^{\left(0\right)}=CI$. A unitary $U\in Q^{\left(k\right)}$ is the exponential of a polynomial of degree k, if $\partial_{\vec{a}}U\in Q^{\left(k-1\right)}$ for all $\vec{a}\in V^{n}$.

The definition of quantum polynomials coincides with the Clifford hierarchy.

Proposition 22 (Algebraic Hierarchy). *For integer* k⩾1, *the unitary exponentials of degree-k polynomials* $Q^{\left(k\right)}$ *equal the unitaries in the* k*-th level* $C^{\left(k\right)}$ *of the Clifford hierarchy*.

Lemma 23. *If* $U\in C^{\left(k\right)}$ *and* $w\left(\vec{a}\right),w\left(\vec{b}\right)$ *are Weyl operators, then* $w\left(a\right)Uw\left(b\right)\in C^{\left(k\right)}$.

***Proof:*** By definition, $C^{\left(1\right)}$ is the group generated by the Weyl operators, so $U\in C^{\left(1\right)}$ ensures that $w\left(a\right)Uw\left(b\right)\in C^{\left(1\right)}$.

We establish this by induction on k. Assume the proposition is valid for $C^{\left(j\right)}$, with j=1,…,k and $U\in C^{\left(k+1\right)}$. Let $W=w\left(\vec{a}\right)Uw\left(\vec{b}\right)$. The induction is valid, if $Ww\left(\vec{c}\right)W^{*}\in C^{\left(k\right)}$ for any $w(\vec{c})$. Note that

$$\begin{array}{c} Ww\left(\vec{c}\right)W^{*}=\omega^{{\langle \vec{b},\vec{c}\rangle }_{s}}\;w\left(\vec{a}\right)Uw\left(\vec{c}\right)U^{*}w{\left(a\right)}^{*}=w\left(\vec{a}\right)Vw{\left(\vec{a}\right)}^{*}\;, \end{array}$$

where $V=\omega^{{\left\langle \vec{b},\vec{c}\right\rangle }_{s}}Uw\left(c\right)U^{*}\in C^{\left(k\right)}$. As we assume $U\in C^{\left(k+1\right)}$, it follows that $V\in C^{\left(k\right)}$. So by the induction hypothesis, also $w\left(\vec{a}\right)Vw{\left(\vec{a}\right)}^{*}\in C^{\left(k\right)}$. Hence, $w\left(\vec{a}\right)Uw\left(\vec{b}\right)\in C^{\left(k+1\right)}$.

***Proof of Proposition 22:*** Let us start with case k=1. Any unitary $U\in Q^{\left(1\right)}$ and any Weyl operator w(a) satisfy $\partial_{\vec{a}}U=w\left(\vec{a}\right)Uw{\left(\vec{a}\right)}^{*}U^{*}\in Q^{\left(0\right)}$. That is $w\left(a\right)Uw{\left(a\right)}^{*}U^{*}=cI$, where c is a complex unit. Thus, $Uw{\left(a\right)}^{*}U=Uw\left(-a\right)U^{*}$ is a Weyl operator, from which we infer that $U\in C^{\left(1\right)}$. Conversely, if $U\in C^{\left(1\right)}$, then U is a Weyl operator w(b). Hence $w\left(a\right)Uw{\left(a\right)}^{*}U^{*}=w\left(a\right)w\left(b\right)w{\left(a\right)}^{*}w{\left(b\right)}^{*}=\omega_{d}^{{\left\langle a,b\right\rangle }_{s}}I$, indicating $U\in Q^{\left(1\right)}$.

We proceed by induction. Assume that $C^{\left(j\right)}=Q^{\left(j\right)}$ for j=1,…,k. Then if $Uw\left(\vec{a}\right)U^{*}\in C^{\left(k\right)}$ for every $w(\vec{a})$, one infers that $U\in C^{\left(k+1\right)}$. On the other hand, if $Uw{\left(\vec{a}\right)}^{*}U^{*}\in C^{\left(k\right)}$, then we infer from *Lemma* 23 that $w\left(\vec{a}\right)Uw{\left(\vec{a}\right)}^{*}U^{*}=\partial_{\vec{a}}U\in C^{\left(k\right)}=Q^{\left(k\right)}$. Hence $U\in Q^{\left(k+1\right)}$.

Conversely, for $U\in Q^{\left(k+1\right)}$, we have $\partial_{\vec{a}}U=w\left(\vec{a}\right)Uw{\left(\vec{a}\right)}^{*}U^{*}=V\in Q^{\left(k\right)}=C^{\left(k\right)}$. Again from *Lemma* 23, we have $Uw{\left(a\right)}^{*}U^{*}=w{\left(a\right)}^{*}V\in C^{\left(k\right)}$. Hence, we have $U\in C^{\left(k+1\right)}$ and $C^{\left(k+1\right)}=Q^{\left(k+1\right)}$.

The first and second levels of the Clifford hierarchy are closed under the adjoint operation; this property does not hold in general. However, some subsets of unitaries are closed under the adjoint operation in any k-th level of Clifford hierarchy.

Corollary 24. *The unitaries generated by degree-k polynomial* $U_{f}=\sum_{\vec{x}}\omega_{d}^{f(\vec{x})}|\vec{x}\rangle \langle \vec{x}|$ *are closed under the adjoint operation in the* k*-th level of Clifford hierarchy*.

## 5. Quantum Norms and Measures

Here we investigate in detail the quantum measures mentioned in Eq. **7**. We use the term “measure” to designate a quantity that in the classical case is a norm; in this paper, we show that these measures are norms for k=2,3.

Definition 25: Given a linear operator B and an integer k⩾1, the quantum uniformity measure ${\left\|\cdot \right\|}_{Q^{k}}$ of order k is defined by Eq. **7**.

We now prove the first inequality in *Theorem* 1.

Proposition 26 (Positivity). *The expression Eq. **7** is nonnegative, so the quantum uniformity measure* ${\|\cdot \|\;}_{Q^{k}}$ *can be taken as its positive root. In particular for* k⩾2,

[26] $$\begin{array}{c} {\left\|B\right\|}_{Q^{k}}^{2^{k}}=E_{{\vec{a}}_{k-1},\ldots ,{\vec{a}}_{1}}{\left|\frac{1}{d^{n}}\mathrm{Tr}\left\{\partial_{{\vec{a}}_{k-1},\cdots ,{\vec{a}}_{1}}B\right\}\right|}^{2}\;, \end{array}$$

*and for* k=1,

[27] $$\begin{array}{c} {\left\|B\right\|}_{Q^{1}}^{2}={\left|\frac{1}{d^{n}}\mathrm{Tr}\left\{B\right\}\right|}^{2}\;. \end{array}$$

***Proof:*** Write for k⩾2,

[28] $$\begin{array}{cc} {\left\|B\right\|}_{Q^{k}}^{2^{k}} & =E_{{\vec{a}}_{k},{\vec{a}}_{k-1},\ldots ,{\vec{a}}_{1}}\frac{1}{d^{n}}\mathrm{Tr}\left\{\partial_{{\vec{a}}_{k}}\partial_{{\vec{a}}_{k-1},\cdots ,{\vec{a}}_{1}}B\right\}\\ & =E_{{\vec{a}}_{k-1},\ldots ,{\vec{a}}_{1}}\frac{1}{d^{n}}\mathrm{Tr}\left\{E_{\vec{a}}\;\left(\partial_{{\vec{a}}_{k-1},\cdots ,{\vec{a}}_{1}}B\right)\left(\vec{a}\right){\left(\partial_{{\vec{a}}_{k-1},\cdots ,{\vec{a}}_{1}}B\right)}^{*}\right\}\\ & =E_{{\vec{a}}_{k-1},\ldots ,{\vec{a}}_{1}}{\left|\frac{1}{d^{n}}\mathrm{Tr}\left\{\partial_{{\vec{a}}_{k-1},\cdots ,{\vec{a}}_{1}}B\right\}\right|}^{2}\;. \end{array}$$

Here we use Eq. **15** to obtain the final equality. In the case k=1, one omits the derivatives ${\vec{a}}_{1},\ldots ,{\vec{a}}_{k-1}$. As an expectation of an absolute squared quantity, the result is nonnegative. See also Eq. **22**.

Proposition 27 (Inductive Property). *The quantum uniformity measures* ${\left\|B\right\|}_{Q^{k}}$ *satisfy the inductive relation*,

[29] $$\begin{array}{c} {\left\|B\right\|}_{Q^{k}}^{2^{k}}=E_{\vec{a}}{\|\partial_{\vec{a}}B\|}_{Q^{k-1}}^{2^{k-1}}\;. \end{array}$$

***Proof:*** Write

[30] $$\begin{array}{cc} \|B{\|}_{Q^{k}}^{2^{k}} & =E_{{\vec{a}}_{1}}E_{{\vec{a}}_{k},\ldots ,{\vec{a}}_{2}}\frac{1}{d^{n}}\text{Tr}\left\{\partial_{{\vec{a}}_{k},\cdots ,{\vec{a}}_{2}}\partial_{{\vec{a}}_{1}}B\right\}\\ & =E_{\vec{a}}{\left\|\partial_{\vec{a}}B\right\|}_{Q^{k-1}}^{2^{k-1}}\;, \end{array}$$

as claimed.

Proposition 28. *The measure* ${\left\|\cdot \right\|}_{Q^{k}}$ *is invariant under conjugation by a Clifford unitary. In other words, for* $U\in C^{\left(2\right)}$,

[31] $$\|UBU^{*}{\|}_{Q^{k}}=\|B{\|}_{Q^{k}}\;.$$

*For* k⩾2, *the measure* ${\left\|\cdot \right\|}_{Q^{k}}$ *is invariant under left or right multiplication by a Weyl operator*,

[32] $$\begin{array}{c} {\|w\left(\vec{a}\right)Bw\left(\vec{b})\right\|}_{Q^{k}}={\left\|B\right\|}_{Q^{k}}\;. \end{array}$$

***Proof:*** For k=1, we infer from *Proposition* 26, that for any unitary U,

$$\begin{array}{c} {\left\|UBU^{*}\right\|}_{Q^{1}}^{2}={\left|\frac{1}{d^{n}}\mathrm{Tr}\left\{UBU^{*}\right\}\right|}^{2}={\left|\frac{1}{d^{n}}\mathrm{Tr}\left\{B\right\}\right|}^{2}={\left\|B\right\|}_{Q^{1}}^{2}\;. \end{array}$$

We proceed by induction on k. Assume that the Eq. **31** holds for ${\left\|\cdot \right\|}_{Q^{k}}$, and consider ${\left\|\cdot \right\|}_{Q^{k+1}}$. Rewrite the quantum derivative $\partial_{\vec{a}}\left(UAU^{*}\right)$ as

$$\begin{array}{cc} \partial_{\vec{a}}\left(UBU^{*}\right) & =w\left(\vec{a}\right)UBU^{*}w{\left(\vec{a}\right)}^{*}UB^{*}U^{*}\\ & =UU^{*}w\left(\vec{a}\right)UBU^{*}w{\left(\vec{a}\right)}^{*}UB^{*}U^{*}\\ & =U\left(\partial_{U^{*}w\left(\vec{a}\right)U}B\right)U^{*}. \end{array}$$

Then, using *Proposition* 27 and the inductive hypothesis, we have

$$\begin{array}{cc} \|UBU^{*}{\|}_{Q^{k+1}}^{2^{k+1}} & =E_{\vec{a}}{\left\|\partial_{\vec{a}}(UBU^{*})\right\|}_{Q^{k}}^{2^{k}}=E_{\vec{a}}{\left\|U(\partial_{U^{*}w(\vec{a})U}B)U^{*}\right\|}_{Q^{k}}^{2^{k}}\\ & =E_{\vec{a}}{\left\|\partial_{U^{*}w(\vec{a})U}B\right\|}_{Q^{k}}^{2^{k}}=E_{\vec{b}}{\left\|\partial_{\vec{b}}B\right\|}_{Q^{k}}^{2^{k}}={\left\|B\right\|}_{Q^{k+1}}^{2^{k+1}}. \end{array}$$

Here we use the fact that conjugation by $U^{*}$ maps $w(\vec{a})$ to another element of the Weyl group, which equals $w(\vec{b})$, up to a phase. Since this map in $Z_{d}$ is 1-to-1, the summation over $\vec{b}$ gives the same result.

To establish Eq. **32**, note that $w\left(\vec{a}\right)Bw\left(\vec{b}\right)=w\left(\vec{a}\right)Bw{\left(\vec{a}\right)}^{*}w\left(\vec{a}+\vec{b}\right)$ up to a phase χ, and ${\left\|B\chi \right\|}_{Q^{k}}={\left\|B\right\|}_{Q^{k}}$ Since $w\left(\vec{a}\right)=U\in C^{\left(1\right)}$, the first statement of the proposition means that we only need to show that ${\left\|Bw(\vec{b})\right\|}_{Q^{k}}={\left\|B\right\|}_{Q^{k}}$. The derivative $\partial_{\vec{a}}\left(Bw\left(\vec{b}\right)\right)$ can be written

[33] $$\begin{array}{c} \partial_{\vec{a}}\left(Bw\left(\vec{b}\right)\right)=\omega^{{\langle \vec{a},\vec{b}\rangle }_{s}}\;B\left(\vec{a}\right)B^{*}=\omega^{{\langle \vec{b},\vec{a}\rangle }_{s}}\;\partial_{\vec{a}}B\;. \end{array}$$

Using *Proposition* 27 and Eq. **33**,

$$\begin{array}{cc} {\left\|Bw(\vec{b})\right\|}_{Q^{k}}^{2^{k}} & =E_{\vec{a}}{\left\|\partial_{\vec{a}}(Bw(\vec{b}))\right\|}_{Q^{k-1}}^{2^{k-1}}\\ & =E_{\vec{a}}{\left\|\omega^{{\langle \vec{b},\vec{a}\rangle }_{s}}\partial_{\vec{a}}B\right\|}_{Q^{k-1}}^{2^{k-1}}\\ & =E_{\vec{a}}{\left\|\partial_{\vec{a}}B\right\|}_{Q^{k-1}}^{2^{k-1}}=\left\|B_{Q^{k}}^{2^{k}}\right\|. \end{array}$$

This completes the proof.

## 6. Schatten and Fourier Norms

Robert Schatten introduced $L^{p}$ norms for linear transformations, which are used widely in analysis. We use the Schatten and Fourier norms on the coefficients $\Xi_{B}\left(\vec{b}\right)=\langle w(\vec{b}),B\rangle$ defined as

$${\left\|B\right\|}_{p}={\left(\frac{1}{d^{n}}\text{Tr}\left\{{\left(B^{*}B\right)}^{p/2}\right\}\right)}^{1/p}\;,\;\;{\left\|{\text{Ξ}}_{B}\right\|}_{l^{p}}^{p}=\sum_{\vec{b}}{\left|{\text{Ξ}}_{B}(\vec{b})\right|}^{p}\;.$$

For p=2 they are related by

[34] $$\begin{array}{c} {\left\|B\right\|}_{2}={\left\|\Xi_{B}\right\|}_{\ell^{2}}\;. \end{array}$$

## 7. Classical Uniformity Norms

Our quantum uniformity measures ${\left\|\cdot \right\|}_{Q^{k}}$ are a generalization of Gowers’ classical uniformity norms ${\left\|\cdot \right\|}_{U^{k}}$; see refs. 1 and 4. For a function $f(\vec{x})$ on $Z_{d}^{n}$, the classical uniformity norm can defined inductively as follows,

$$\begin{array}{c} {\left\|f\right\|}_{U^{k}}^{2^{k}}=E_{\vec{q}}{\left\|\partial_{\vec{q}}f\right\|}_{U^{k-1}}^{2^{k-1}}\;,\;\;\text{and}\;{\left\|f\right\|}_{U^{1}}=\left|\frac{1}{d^{n}}\sum_{\vec{x}\in Z_{d}^{n}}f\left(\vec{x}\right)\right|\;. \end{array}$$

For transformations B that are diagonal in the computational basis, the quantum uniformity measures reduce to the classical norms.

Proposition 29 (Reduction to Gowers’ Norms). *Let* $B_{f}$ *have the form Eq. **23**, and let* k⩾1. *Then*

[35] $$\begin{array}{c} \|Bf{\|}_{Q^{k}}=\|f{\|}_{U^{k}}\;. \end{array}$$

***Proof:*** The identity Eq. **35** holds for k=1 as a consequence of *Proposition* 26 and calculation of the trace in the computational basis. We proceed by induction on k. Assume Eq. **35** holds for j=1,…,k−1. Then using *Proposition* 20 to compute $\partial_{\vec{a}}B_{f}$, one has

$$\begin{array}{cc} {\left\|B_{f}\right\|}_{Q^{k}}^{2^{k}} & =E_{\vec{a}}{\left\|\partial_{\vec{a}}B_{f}\right\|}_{Q^{k-1}}^{2^{k-1}}=E_{\vec{a}}{\left\|B_{\partial_{-\vec{q}}f}\right\|}_{Q^{k-1}}^{2^{k-1}}={\left\|f\right\|}_{U^{k}}^{2^{k}}\;. \end{array}$$

In the last equality we use the inductive hypothesis.

## 8. Examples of Some Quantum Uniformity Measures

Here we give the quantum uniformity measures for several gates, with details in *SI Appendix*. We consider qudit systems with local dimension equal 2 or an odd prime. Observe these quantum uniformity measures increase monotonically in k, as proved later in *Theorem* 38.

Example 30 (Weyl Gates): The Weyl (or Pauli) gates are given by the Weyl operators $w(\vec{b})$. Their quantum uniformity norms are

$$\begin{array}{c} \begin{array}{c} {\left\|w(\vec{b})\right\|}_{Q^{1}}=\delta_{\vec{b},\vec{0}}\;,\;{\left\|w(\vec{b})\right\|}_{Q^{k}}=1,\;\forall k⩾2\;. \end{array} \end{array}$$

Example 31 (The Fourier Gate): For d=2, this gate is called the Hadamard gate. The Fourier gate for an n-qudit system of dimension d is $F=\frac{1}{d^{n/2}}\sum_{x,y\in {\mathbb{Z}}_{d}^{n}}\omega^{\vec{x}\cdot \vec{y}}|\vec{x}\rangle \langle \vec{y}|$.

$$\begin{array}{cc} {\left\|F\right\|}_{Q^{1}} & =\left\{\begin{array}{cc} 0\;, & \text{if}\;\;d=2\\ \frac{1}{d^{n}}\;, & \text{if d is odd prime} \end{array}\right.\;,\\ {\left\|F\right\|}_{Q^{2}} & =\left\{\begin{array}{cc} \frac{1}{2^{n/4}}, & \text{if}\;\;d=2\\ \frac{1}{d^{n/2}}, & \text{if d is odd prime} \end{array}\right.\;,\\ {\left\|F\right\|}_{Q^{k}} & =1,\;\forall k⩾3\;. \end{array}$$

Example 32 (The CNOT Gate): The two-qudit “control-not gate” plays an important role in generating quantum entanglement.

[36] $$\begin{array}{c} CNOT=\sum_{x,y\in {\mathbb{Z}}_{d}}|x\rangle \langle x|⊗|x+y\rangle \langle y|. \end{array}$$

For both d=2 or d= odd prime,

$$\begin{array}{cc} {\left\|\mathrm{CNOT}\right\|}_{Q^{1}} & =\frac{1}{d}\;,{\left\|\mathrm{CNOT}\right\|}_{Q^{2}}=\frac{1}{\sqrt{d}},\\ {\left\|\mathrm{CNOT}\right\|}_{Q^{k}} & =1,\;\forall k⩾3\;. \end{array}$$

Example 33 (The CCZ Gate): The three-qudit “control–control-Z gate,” or CCZ, in the computational basis is

[37] $$\begin{array}{c} CCZ=\sum_{x,y,z}\omega_{d}^{\mathrm{xyz}}\left|x\rangle \langle x\right|⊗\left|y\rangle \langle y\right|⊗|z\rangle \langle z|. \end{array}$$

The CCZ gate also plays an important role in the theory of universal quantum computation. For d an odd prime,

$$\begin{array}{cc} & {\left\|\mathrm{CCZ}\right\|}_{Q^{1}}=\frac{2d-1}{d^{2}}\\ & {\left\|\mathrm{CCZ}\right\|}_{Q^{2}}={\left(\frac{d^{3}+{\left(d-1\right)}^{3}+d\left[d^{3}-{\left(d-1\right)}^{3}-1\right]}{d^{6}}\right)}^{1/4},\\ & {\left\|\mathrm{CCZ}\right\|}_{Q^{3}}={\left(\frac{d^{3}+3\left(d-1\right)d+3{\left(d-1\right)}^{2}d+{\left(d-1\right)}^{3}}{d^{6}}\right)}^{1/8},\\ & {\left\|\mathrm{CCZ}\right\|}_{Q^{k}}=1,\forall k⩾4 \end{array}$$

If d=2, the corresponding quantum uniformity norms are

$$\begin{array}{cc} & {\left\|\mathrm{CCZ}\right\|}_{Q^{1}}=\frac{3}{4},{\left\|\mathrm{CCZ}\right\|}_{Q^{2}}={\left(\frac{11}{32}\right)}^{1/4},\\ & {\left\|\mathrm{CCZ}\right\|}_{Q^{3}}={\left(\frac{11}{32}\right)}^{1/8},{\left\|\mathrm{CCZ}\right\|}_{Q^{k}}=1,\;\;\forall k⩾4\;. \end{array}$$

## 9. More Properties of the Quantum Uniformity Measures

We explore more mathematical properties of the quantum uniformity measures, including: monotonicity of ${\left\|\cdot \right\|}_{Q^{k}}$ with respect to k, proving ${\left\|\cdot \right\|}_{Q^{k}}$ is a norm for k=2,3, and showing relations between ${\left\|\cdot \right\|}_{Q^{k}}$ and both Schatten and $\ell^{p}$ norms.

Given $2^{k}$ linear operators ${\left\{B_{\vec{u}}\right\}}_{\vec{u}\in 0,1^{k}}$, with binary labels $\vec{u}$, we define ${\left\{B_{\vec{u}}\right\}}_{\vec{u}\in {\left\{0,1\right\}}^{k}}({\vec{a}}_{k},..,{\vec{a}}_{1})$ inductively. For k=1,

$$\{B_{\vec{u}}{\}}_{\vec{u}\in {\left\{0,1\right\}}^{1}}({\vec{a}}_{1})=\left\{B_{0},B_{1}\right\}({\vec{a}}_{1}):=B_{0}({\vec{a}}_{1})B_{1}^{*}.$$

This is chosen so that if $B_{0}=B_{1}=B$, then ${\left\{B_{\vec{u}}\right\}}_{\vec{u}\in {\left\{0,1\right\}}^{1}}(\vec{a})=\partial_{\vec{a}}B$. For k=2 we use the two sets,

$$\begin{array}{cc} {\left\{B_{0\vec{u}}\right\}}_{\vec{u}\in {\left\{0,1\right\}}^{1}}({\vec{a}}_{1}) & =\left\{B_{00},B_{01}\right\}({\vec{a}}_{1})\;,\\ {\left\{B_{1\vec{u}}\right\}}_{\vec{u}\in \left\{0,1^{1}\right\}}({\vec{a}}_{1}) & =\left\{B_{10},B_{11}\right\}({\vec{a}}_{1})\;, \end{array}$$

giving

$$\begin{array}{cc} & {\left\{B_{\vec{u}}\right\}}_{\vec{u}\in {\left\{0,1\right\}}^{2}}({\vec{a}}_{2},{\vec{a}}_{1})\\ & =\left\{B_{00},B_{01},B_{10},B_{11}\right\}({\vec{a}}_{2},{\vec{a}}_{1})\\ & =w({\vec{a}}_{2})\left(\left\{B_{0\vec{u}}\right\}({\vec{a}}_{1})\right)w{\left({\vec{a}}_{2}\right)}^{*}{\left(\left\{B_{1\vec{u}}\right\}({\vec{a}}_{1})\right)}^{*}. \end{array}$$

Definition 34: Given ${\left\{B_{\vec{u}}\right\}}_{\vec{u}\in {\left(0,1\right)}^{1}}({\vec{a}}_{1})$ above, for k⩾1 let

[38] $$\begin{array}{cc} & {\left\{B_{0\vec{u}}\right\}}_{\vec{u}\in {\left\{0,1\right\}}^{k}}\left({\vec{a}}_{k},\ldots ,{\vec{a}}_{1}\right)\\ & =\left\{B_{000\cdots 00},B_{000\cdots 01},\cdots ,B_{011\cdots 11}\right\}\left({\vec{a}}_{k},\ldots ,{\vec{a}}_{1}\right)\;, \end{array}$$

[39] $$\begin{array}{cc} & {\left\{B_{1\vec{u}}\right\}}_{\vec{u}\in {\left\{0,1\right\}}^{k}}\left({\vec{a}}_{k},\ldots ,{\vec{a}}_{1}\right)\\ & =\left\{B_{100\cdots 00},B_{100\cdots 01},\cdots ,B_{111\cdots 11}\right\}\left({\vec{a}}_{k},\ldots ,{\vec{a}}_{1}\right)\;, \end{array}$$

$$\begin{array}{cc} & {\left\{B_{\vec{u}}\right\}}_{\vec{u}\in {\left\{0,1\right\}}^{k+1}}({\vec{a}}_{k+1},..,{\vec{a}}_{1})\\ & :=w({\vec{a}}_{k+1})\left({\left\{B_{0,\vec{u}}\right\}}_{\vec{u}\in {\left\{0,1\right\}}^{k}}({\vec{a}}_{k},..,{\vec{a}}_{1})\right)w{\left({\vec{a}}_{k+1}\right)}^{*}\\ & \times {\left({\left\{B_{1,\vec{u}}\right\}}_{\vec{u}\in {\left\{0,1\right\}}^{k}}({\vec{a}}_{k},..,{\vec{a}}_{1})\right)}^{*}. \end{array}$$

The quantity ${\left\{B_{\vec{u}}\right\}}_{\vec{u}\in {\left\{0,1\right\}}^{k}}({\vec{a}}_{k},..,{\vec{a}}_{1})$ is a generalization of the k-th order quantum derivative. We use these quantities to define a quantum uniformity functional and a quantum uniformity inner product.

Definition 35 (Quantum Uniformity Functionals): Given ${B_{\vec{u}}}_{\vec{u}\in {0,1}^{k+1}}$ with $2^{k+1}$ linear operators, the quantum uniformity inner functionals of (k+1)th order are

[40] $$\begin{array}{cc} & {\langle {\left\{B_{\vec{u}}\right\}}_{\vec{u}\in 0,1^{k+1}}\rangle }_{Q^{k+1}}\\ & :=E_{{\vec{a}}_{k+1},...{\vec{a}}_{1}\in V^{n}}\frac{1}{d^{n}}\text{Tr}\left\{{\left\{B_{\vec{u}}\right\}}_{\vec{u}\in 0,1^{k+1}}({\vec{a}}_{k+1},..,{\vec{a}}_{1})\right\}\;. \end{array}$$

If all the operators in the set $\left\{B_{\vec{u}}\right\}$ are equal B, then the quantum uniformity functional reduces to the quantum uniformity measures ${\langle {\left\{B_{\vec{u}}\right\}}_{\vec{u}\in 0,1^{k}}\rangle }_{Q^{k}}=\|B{\|}_{Q^{k}}^{2^{k}}$.

Definition 36: Given two sets ${\left\{B_{0{\vec{u}}_{k}}\right\}}_{{\vec{u}}_{k}\in {\left\{0,1\right\}}^{k}}$ and ${\left\{B_{1{\vec{u}}_{k}}\right\}}_{{\vec{u}}_{k}\in {\left\{0,1\right\}}^{k}}$ of $2^{k}$ operators on $H^{⊗n}$, we regard them as subsets of ${\left\{B_{{\vec{u}}_{k+1}}\right\}}_{{\vec{u}}_{k+1}\in {\left\{0,1\right\}}^{k+1}}$. The functional ${\langle \;\cdot \;\rangle }_{Q^{k+1}}$ determines a pairing ${\langle \;\cdot \;,\;\cdot \;\rangle }_{Q^{k}}$ of these subsets.

For simplicity of notation, let us not explicitly note that ${\vec{u}}_{k}\in {\left(0,1\right)}^{k}$. Then the pairing is

[41] $$\langle \left\{B_{0{\vec{u}}_{k}}\right\},\left\{B_{1{\vec{u}}_{k}}\right\}\rangle_{Q^{k}}={\langle \left\{B_{{\vec{u}}_{k+1}}\right\}\rangle }_{Q^{k+1}}.$$

Proposition 37 (Inner Product). *With the notation introduced in Definition 36, the pairing Eq. **41** is a preinner product. It is a nonnegative, hermitian form that is linear in the first variable, conjugate linear in the second variable. It can be written*

[42] $$\begin{array}{l} {\langle \left\{B_{0{\vec{u}}_{k}}\right\},\left\{B_{1{\vec{u}}_{k}}\right\}\rangle }_{Q^{k}}={\langle \left\{B_{{\vec{u}}_{k+1}}\right\}\rangle }_{Q^{k+1}}\\ =\underset{{\vec{a}}_{1},...,{\vec{a}}_{k-1}}{E}\frac{1}{d^{2n}}\left(\text{Tr}\left\{B_{0{\vec{u}}_{k}}({\vec{a}}_{k},..,{\vec{a}}_{1})\right\}\right) \end{array}$$

[43] $$\times \bar{\left(\text{Tr}\left\{B_{1{\vec{u}}_{k}}({\vec{a}}_{k},..,{\vec{a}}_{1})\right\}\right)}.$$

*The pairing satisfies the Schwarz inequality,*

[44] $$\begin{array}{cc} & \left|{\langle \left\{B_{0\vec{u}}\right\},\left\{B_{1\vec{u}}\right\}\rangle }_{Q^{k}}\right|\\ ⩽ & {\langle \left\{B_{0\vec{u}}\right\},\left\{B_{0\vec{u}}\right\}\rangle }_{Q^{k}}^{\frac{1}{2}}{\langle \left\{B_{1\vec{u}}\right\},\left\{B_{1\vec{u}}\right\}\rangle }_{Q^{k}}^{\frac{1}{2}}\;. \end{array}$$

***Proof:*** Using Eq. **15**, we have

[45] $$\begin{array}{cc} & {\langle {\left\{B_{0\vec{u}}\right\}}_{u\in {\left\{0,1\right\}}^{k}},{\left\{B_{1\vec{u}}\right\}}_{u\in {\left\{0,1\right\}}^{k}}\rangle }_{Q^{k}}={\langle \left\{B_{{\vec{u}}_{k+1}}\right\}\rangle }_{Q^{k+1}}\\ & =\underset{{\vec{a}}_{k},...{\vec{a}}_{1}\in V^{n}}{E}\frac{1}{d^{n}}\text{Tr}\left\{{\left\{B_{0\vec{u}}\right\}}_{\vec{u}\in {\left\{0,1\right\}}^{k}}({\vec{a}}_{k},..,{\vec{a}}_{1})\right\}\\ & \times \bar{\frac{1}{d^{n}}\text{Tr}\left\{{\left\{B_{1\vec{u}}\right\}}_{\vec{u}\in {\left\{0,1\right\}}^{k}}({\vec{a}}_{k},..,{\vec{a}}_{1})\right\}}\;. \end{array}$$

From this representation, one can see the pairing has the correct linearity properties. Furthermore, this shows that the pairing is hermitian,

[46] $$\begin{array}{c} {\langle \left\{B_{0{\vec{u}}_{k}}\right\},\left\{B_{1{\vec{u}}_{k}}\right\}\rangle }_{Q^{k}}={\bar{\langle \left\{B_{1{\vec{u}}_{k}}\right\}\rangle ,\left\{B_{0{\vec{u}}_{k}}\right\}}}_{Q^{k}}. \end{array}$$

If $\left\{B_{0\vec{u}}\right\}=\left\{B_{1\vec{u}}\right\}$, then each term in the expectation is nonnegative, so the expectation is nonnegative. To obtain Eq. **44**, use the Schwarz inequality on the sequences in Eq. **42**.

Note that the expression Eq. **45** is not strictly positive; for if k=1, and $B_{00}=B_{10}=w\left(\vec{b}\right)$, $B_{01}=B_{11}=w\left(\vec{c}\right)$ with $\vec{b}\neq \vec{c}$, then Eq. **45** is zero. In particular

[47] $$\begin{array}{cc} 0 & ⩽{\langle {\left\{B_{0\vec{u}}\right\}}_{u\in {\left(0,1\right)}^{k}},{\left\{B_{0\vec{u}}\right\}}_{u\in {\left(0,1\right)}^{k}}\rangle }_{Q^{k}}\\ & =E_{\vec{a}}\frac{1}{d^{2n}}{\left|\text{Tr}\left\{w(\vec{a})w(\vec{b})w{\left(\vec{a}\right)}^{*}w{\left(\vec{c}\right)}^{*}\right\}\right|}^{2}=\delta_{\vec{b},\vec{c}}=0\;. \end{array}$$

However, the form is strictly positive if all the B’s are the same.

Taking *Proposition* 37 into account, one can define

[48] $$\|{\left\{B_{0\vec{u}}\right\}}_{u\in 0,1^{k}}{\|}_{Q^{k}}={\langle {\left\{B_{0\vec{u}}\right\}}_{u\in {\left\{0,1\right\}}^{k}},{\left\{B_{0\vec{u}}\right\}}_{u\in {\left(0,1\right)}^{k}}\rangle }_{Q^{k}}^{\frac{1}{2}}\;,$$

as the right-hand side is nonnegative. Similarly

[49] $$\|{\left\{B_{1\vec{u}}\right\}}_{u\in {\left\{0,1\right\}}^{k}}{\|}_{Q^{k}}={\langle {\left\{B_{1\vec{u}}\right\}}_{u\in {\left\{0,1\right\}}^{k}},{\left\{B_{1\vec{u}}\right\}}_{u\in 0,1^{k}}\rangle }_{Q^{k}}^{\frac{1}{2}}\;.$$

Theorem 38 (Monotonocity of Measures). *For any* n*-qudit linear operator* $B\in L(H^{⊗n})$, *the measures Eq. **7** satisfy*

[50] $$\begin{array}{c} {\left\|B\right\|}_{Q^{k}}⩽{\left\|B\right\|}_{Q^{k+1}},\;\;\mathrm{for all}\;k⩾1. \end{array}$$

***Proof:*** Choose ${\left\{B_{0}\right\}}_{\vec{u}\in {\left\{0,1\right\}}^{1}}=B$ and ${\left\{B_{1}\right\}}_{\vec{u}\in {\left\{0,1\right\}}^{1}}=\left\{I\right\}$. Then *Proposition* 37 yields the desired bound.

We now illustrate some further properties of ${\left\|\cdot \right\|}_{Q^{k}}$ for k=2,3.

Proposition 39. *For* k=2 *one has*

$$\begin{array}{cc} {\langle B_{00},B_{01},B_{10},B_{11}\rangle }_{Q^{2}} & =\sum_{\vec{b}}{\text{Ξ}}_{B_{00}}(\vec{b})\bar{{\text{Ξ}}_{B_{01}}(\vec{b})}\bar{{\text{Ξ}}_{B_{10}}(\vec{b})}{\text{Ξ}}_{B_{11}}(\vec{b}).\\ \left|{\langle B_{00},B_{01},B_{10},B_{11}\rangle }_{Q^{2}}\right| & ⩽\prod_{i,j=0}^{1}{\left\|B_{ij}\right\|}_{Q^{2}}. \end{array}$$

*Also* ${\left\|B\right\|}_{Q^{2}}={\left\|\text{Ξ}\right\|}_{Bl^{4}}$, *and* ${\left\|\cdot \right\|}_{Q^{2}}$ *is a norm*.

***Proof:*** As $T^{*}=\sum_{\vec{b}}\bar{\Xi_{T}\left(\vec{b}\right)}\;w\left(-\vec{b}\right)$, it follows that

$$\begin{array}{cc} & {\langle B_{00},B_{01},B_{10},B_{11}\rangle }_{Q^{2}}\\ & =E_{{\vec{a}}_{1},{\vec{a}}_{2}}\frac{1}{d^{n}}\mathrm{Tr}\left\{\left(B_{00}\left({\vec{a}}_{1}\right)B_{01}^{*}\right)\left({\vec{a}}_{2}\right){\left(B_{10}\left({\vec{a}}_{1}\right)B_{11}^{*}\right)}^{*}\right\}\\ & =E_{{\vec{a}}_{1},{\vec{a}}_{2}}\frac{1}{d^{n}}\sum_{{\vec{b}}_{1},{\vec{b}}_{2},{\vec{b}}_{3},{\vec{b}}_{4}}\omega^{{\langle {\vec{a}}_{1}+{\vec{a}}_{2},{\vec{b}}_{1}\rangle }_{s}-{\langle {\vec{a}}_{2},{\vec{b}}_{2}\rangle }_{s}-{\langle {\vec{a}}_{1},{\vec{b}}_{3}\rangle }_{s}}\\ & \times \Xi_{B_{00}}\left({\vec{b}}_{1}\right)\bar{\Xi_{B_{01}}\left({\vec{b}}_{2}\right)}\;\bar{\Xi_{B_{10}}\left({\vec{b}}_{3}\right)}\Xi_{B_{11}}\left({\vec{b}}_{4}\right)\\ & \times \mathrm{Tr}\left\{w\left({\vec{b}}_{1}\right)w\left(-{\vec{b}}_{2}\right)w\left(-{\vec{b}}_{3}\right)w\left({\vec{b}}_{4}\right)\right\}\\ & =\sum_{{\vec{b}}_{1},{\vec{b}}_{2},{\vec{b}}_{3},{\vec{b}}_{4}}\delta_{{\vec{b}}_{1},{\vec{b}}_{3}}\delta_{{\vec{b}}_{1},{\vec{b}}_{2}}\delta_{{\vec{b}}_{1}-{\vec{b}}_{2},{\vec{b}}_{3}-{\vec{b}}_{4}}\\ & \times \Xi_{B_{00}}\left({\vec{b}}_{1}\right)\bar{\Xi_{B_{01}}\left({\vec{b}}_{2}\right)}\;\bar{\Xi_{B_{10}}\left({\vec{b}}_{3}\right)}\Xi_{B_{11}}\left({\vec{b}}_{4}\right)\\ & =\sum_{\vec{b}}\Xi_{B_{00}}\left(\vec{b}\right)\bar{\Xi_{B_{01}}\left(\vec{b}\right)}\;\bar{\Xi_{B_{10}}\left(\vec{b}\right)}\Xi_{B_{11}}\left(\vec{b}\right)\;. \end{array}$$

From this identity and Hölder’s inequality, we infer the upper bound. Setting the B’s equal, one has $\|B{\|}_{Q^{2}}=\|{\text{Ξ}}_{B}{\|}_{l^{4}}$. Since $\Xi_{B}$ is linear in B and $\|{\text{Ξ}}_{B}{\|}_{l^{4}}$ is a norm, also ${\left\|B\right\|}_{Q^{2}}$ is a norm.

For the case where k=3, the relation to $\ell^{p}$ norms in Fourier space is more complicated; one has several independent vector summations, rather than only 1 for k=2. In order to obtain a generalized Schwarz inequality, we establish the following.

Lemma 40. *Given 8 linear operators* ${\left\{B_{\vec{u}}\right\}}_{\vec{u}\in {\left\{0,1\right\}}^{3}}$, *we have two permutation identities:*

[51] $$\begin{array}{cc} & {\left\langle B_{000},B_{001},B_{010},B_{011},B_{100},B_{101},B_{110},B_{111}\right\rangle }_{Q^{3}}\\ & ={\left\langle B_{010}^{*},B_{000}^{*},B_{011}^{*},B_{001}^{*},B_{110}^{*},B_{100}^{*},B_{111}^{*},B_{101}^{*}\right\rangle }_{Q^{3}}, \end{array}$$

*and*

$$\begin{array}{cc} & {\left\langle B_{000},B_{001},B_{010},B_{011},B_{100},B_{101},B_{110},B_{111}\right\rangle }_{Q^{3}}\\ & ={\left\langle B_{011},B_{010},B_{111},B_{110},B_{001},B_{000},B_{101},B_{100}\right\rangle }_{Q^{3}}. \end{array}$$

***Proof:*** To simplify notation, denote $T({\vec{a}}_{1})$ by T(1). To establish the first equality, use *Proposition* 37 to obtain

$$\begin{array}{cc} & {\left\langle B_{000},B_{001},B_{010},B_{011},B_{100},B_{101},B_{110},B_{111}\right\rangle }_{Q^{3}}\\ & =E_{2,1}\frac{1}{d^{2n}}\mathrm{Tr}\left\{B_{000}\left(1+2\right)B_{001}^{*}\left(2\right)B_{011}B_{010}^{*}\left(1\right)\right\}\\ & \times \bar{\mathrm{Tr}\left\{B_{100}\left(1+2\right)B_{101}^{*}\left(2\right)B_{111}B_{110}^{*}\left(1\right)\right\}}\;. \end{array}$$

Use cyclicity of the trace and translation by $-{\vec{a}}_{2}$ to rewrite the first trace as $\mathrm{Tr}\left\{B_{010}^{*}\left(1-2\right)B_{000}\left(1\right)B_{001}^{*}B_{011}\left(-2\right)\right\}$. Make the same manipulation of the second trace. The expectation over ${\vec{a}}_{2}$ equals the expectation over $-{\vec{a}}_{2}$. Also one can interchange the expectation over ${\vec{a}}_{1}$ and ${\vec{a}}_{2}$. This gives the desired identity. In order to establish the second equality, note that

$$\begin{array}{c} {\langle B_{000},B_{001},B_{010},B_{011},B_{100},B_{101},B_{110},B_{111}\rangle }_{Q^{3}}\\ =E_{3,2,1}\frac{1}{d^{n}}\text{Tr}\{B_{000}(1+2+3)B_{001}^{*}(2+3)B_{011}(3)\\ \times B_{010}^{*}(1+3)B_{110}(1)B_{111}^{*}B_{101}(2)B_{100}(1+2)\}. \end{array}$$

One completes the proof in the same manner as was used to establish the first identity.

Proposition 41 (Generalized Quantum Schwarz Inequality). Let k=3 and let ${\left\{B_{\vec{u}}\right\}}_{\vec{u}\in {\left\{0,1\right\}}^{k}}$ be 8 linear operators. Then,

[52] $$\begin{array}{c} |{\langle {\left\{B_{\vec{u}}\right\}}_{\vec{u}\in {\left\{0,1\right\}}^{k}}\rangle }_{Q^{k}}|⩽{\text{Π}}_{\vec{u}\in {\left\{0,1\right\}}^{k}}{\left\|B_{\vec{u}}\right\|}_{Q^{k}}. \end{array}$$

The proof is in *SI Appendix*.

Theorem 42. *For* k=3, *the* ${\left\|\cdot \right\|}_{Q^{k}}$ *defines a norm*.

***Proof:*** A norm has four properties: positivity, scaling, nondegeneracy, and the triangle inequality. Positivity for all k was established in *Proposition* 26. Scaling for all k is clear from the definition. Nondegeneracy is true as k=2 gives a norm and the monotonicity property established in *Theorem* 38. Thus we only need to prove the triangle inequality, which we prove for k=3.

$$\begin{array}{cc} \|B_{0}+B_{1}{\|}_{Q^{k}}^{2^{k}} & ={\langle {\left\{B_{0}+B_{1}\right\}}_{\vec{u}\in 0,1^{k}}\rangle }_{Q^{k}}\\ & =\sum_{S\subset 0,1^{k}}{\langle {\left\{B_{I_{\vec{u}\in S}}\right\}}_{\vec{u}\in 0,1^{k}}\rangle }_{Q^{k}}\\ & ⩽\sum_{S\subset 0,1^{k}}{\text{Π}}_{\vec{u}\in 0,1^{n}}\|B_{I_{\vec{u}\in S}}{\|}_{Q^{k}}\\ & ={\left(\|B_{0}{\|}_{Q^{k}}+\|B_{1}{\|}_{Q^{k}}\right)}^{2^{k}}\;. \end{array}$$

Here $I_{\vec{u}\in S}$ is the indicator function for the subset S that chooses $B_{0}$ in the set S and $B_{1}$ in its complement. We have used the upper bound in *Proposition* 41 for each term with k=3. Hence, we have the triangle inequality ${\left\|B_{0}+B_{1}\right\|}_{Q^{k}}⩽{\left\|B_{0}\right\|}_{Q^{k}}+{\left\|B_{1}\right\|}_{Q^{k}}$ in case that k=3.

Remark 43: It would be interesting to show whether ${\left\|\cdot \right\|}_{Q^{k}}$ satisfies the triangle inequality for k⩾4.

In the classical theory of uniformity norms, the connection between $L^{p}$ norms and Gowers’ norms has been studied by Eisner and Tao (46). In this work, we investigate the connection between the quantum uniformity measure ${\left\|\cdot \right\|}_{Q^{k}}$ (or norm with k=2,3) and the $L^{p}$ norm.

Lemma 44. *Given two positive operators* $A,B\in L(H^{⊗n})$ *and integer* k⩾0, *one has*

[53] $$\begin{array}{c} 0⩽E_{\vec{a}\in V^{n}}\frac{1}{d^{n}}\mathrm{Tr}\left\{{\left(A(\vec{a})\;B\right)}^{k}\right\}⩽{\left\|A\right\|}_{k}^{k}{\left\|B\right\|}_{k}^{k}. \end{array}$$

***Proof:*** Note that ${\left(AB\right)}^{k}=AB^{1/2}{\left(B^{1/2}AB^{1/2}\right)}^{k-1}B^{1/2}$, so

$$\mathrm{Tr}\left\{{\left(AB\right)}^{k}\right\}=\mathrm{Tr}\left\{{\left(B^{1/2}AB^{1/2}\right)}^{k}\right\}⩾0\;.$$

The Araki-Lieb-Thirring inequality (47, 48) states that for positive operators A,B and positive integer k,

$$\begin{array}{c} 0⩽\mathrm{Tr}\left\{{\left(AB\right)}^{k}\right\}⩽\mathrm{Tr}\left\{A^{k}B^{k}\right\}\;. \end{array}$$

As $A\mapsto A(\vec{a})$ preserves positivity,

$$\begin{array}{cc} 0⩽E_{\vec{a}\in V^{n}}\frac{1}{d^{n}}\mathrm{Tr}\left\{{\left(A\left(\vec{a}\right)B\right)}^{k}\right\} & ⩽E_{\vec{a}\in V^{n}}\frac{1}{d^{n}}\mathrm{Tr}\left\{A^{k}\left(\vec{a}\right)B^{k}\right\}\;. \end{array}$$

Using Eq. **15**, one has

$$\begin{array}{cc} E_{\vec{a}}\frac{1}{d^{n}}\mathrm{Tr}\left\{A^{k}\left(\vec{a}\right)B^{k}\right\} & =\left(\frac{1}{d^{n}}\mathrm{Tr}\left\{A^{k}\right\}\right)\left(\frac{1}{d^{n}}\mathrm{Tr}\left\{B^{k}\right\}\right)={\left\|A\right\|}_{k}^{k}{\left\|B\right\|}_{k}^{k}\;. \end{array}$$

Therefore, we obtain the result.

Proposition 45. *Given two operators* $A,B\in L(H^{⊗n})$ *and an integer* k⩾1, *we have*

[54] $$\begin{array}{c} {\left(E_{\vec{a}\in V^{n}}{\left\|A\left(\vec{a}\right)B\right\|}_{r}^{s}\right)}^{1/s}⩽{\left\|A\right\|}_{p}{\left\|B\right\|}_{q}, \end{array}$$

*where* $p=q=2^{k}/\left(k+1\right)$, $r=2^{k-1}/k$, *and* $s=2^{k-1}$.

We prove *Proposition* 45 in *SI Appendix*.

Theorem 46 (Schatten Norms Dominate Quantum Uniformity). *For integer* k⩾1 *and* $B\in L(H^{⊗n})$,

$${\left\|B\right\|}_{Q^{k}}⩽{\left\|B\right\|}_{p_{k}}\;,\;\;\mathrm{where}\;p_{k}=\frac{2^{k}}{k+1}\;.$$

***Proof:*** When k=1, we use Eq. **15** to infer that

$$\begin{array}{c} {\left\|B\right\|}_{Q^{1}}^{2}=E_{\vec{a}\in V^{n}}\frac{1}{d^{n}}\mathrm{Tr}\left\{B\left(\vec{a}\right)B^{*}\right\}={\left|\frac{1}{d^{n}}\mathrm{Tr}\left\{B\right\}\right|}^{2}⩽{\left\|B\right\|}_{1}^{2}. \end{array}$$

Here we also use the well-known bound $\left|\frac{1}{d^{n}}\mathrm{Tr}\left\{B\right\}\right|⩽{\left\|B\right\|}_{1}$.

For k>1 we proceed inductively. Assume the statement holds for k=1,…,K. For the k+1 case, we have

$$\begin{array}{c} {\left\|A\right\|}_{Q^{k+1}}^{2^{k+1}}=E_{\vec{a}\in V^{n}}\|\partial_{\vec{a}}{A\|}_{Q^{k}}^{2^{k}}⩽E_{\vec{a}\in V^{n}}\|A\left(\vec{a}\right)A^{†}{\|}_{p_{k}}^{2^{k}}⩽{\left\|A\right\|}_{p_{k+1}}^{2^{k+1}}, \end{array}$$

where the first inequality used the inductive assumption, and the second used *Proposition* 45.

Proposition 47 (Pure States). *Consider any* n*-qudit, pure state* $\rho =\left|\psi \rangle \langle \psi \right|=\sum_{\vec{a}\in V^{n}}\Xi_{\rho }\left(\vec{a}\right)\;w\left(\vec{a}\right)$. *Then*

$$\begin{array}{ll} \|\rho {\|}_{Q^{1}}=\frac{1}{d^{n}}; & \;\;\;\;\;\;\|\rho {\|}_{Q^{2}}=\|\text{Ξ}{\|}_{\rho_{l^{4}}};\\ \|\rho {\|}_{Q^{3}}=d^{n/4}\|{\text{Ξ}}_{\rho }{\|}_{l^{4}}; & \;\;\;\;\;\;\;{\left\|\rho \right\|}_{Q^{4}}=d^{n/2}\|{\text{Ξ}}_{\rho }{\|}_{l^{8}}^{1/2}\|{\text{Ξ}}_{\rho }{\|}_{l^{4}}^{1/2} \end{array}$$

*In general for* k⩾3, *the* $Q^{k}$ *measure of a pure state* ρ *is given by* $\ell^{2^{p}}$ *norms of its Fourier coefficients* $\Xi_{\rho }\left(\vec{a}\right)$ *for* 2⩽p⩽k−1.

[55] $$\|\rho {\|}_{Q^{k}}=d^{n\left(1-\frac{2k}{2^{k}}\right)}{\left\|{\text{Ξ}}_{\rho }\right\|}_{l^{4}}^{2^{-k+2}}\prod_{j=0}^{k-3}{\left\|{\text{Ξ}}_{\rho }\right\|}_{l^{2^{\left(j+2\right)}}}^{2^{-k+j+2}}\;.$$

***Proof:*** For k=1,2 use *Proposition* 2. For k⩾3, we have

$$\begin{array}{ll} \|\rho {\|}_{Q^{k}}^{2^{k}} & =E_{\vec{a}}\|\partial_{\vec{a}}\rho {\|}_{Q^{k-1}}^{2^{k-1}}\\ & =E_{\vec{a}}|\langle \psi |w{\left(\vec{a}\right)}^{*}|\rangle \psi {|}^{2^{k-1}}\|w(\vec{a})\rho {\|}_{Q^{k-1}}^{2^{k-1}},\\ & =E_{\vec{a}}d^{n2^{k-1}}|{\text{Ξ}}_{\rho }(\vec{a}){|}^{2^{k-1}}\|w(\vec{a})\rho {\|}_{Q^{k-1}}^{2^{k-1}},\\ & =E_{\vec{a}}d^{n2^{k-1}}|{\text{Ξ}}_{\rho }(\vec{a}){|}^{2^{k-1}}\|\rho {\|}_{Q^{k-1}}^{2^{k-1}},\\ & =d^{n(2^{k-1}-2)}\|{\text{Ξ}}_{\rho }{\|}_{l^{2^{k-1}}}^{2^{k-1}}\|\rho {\|}_{Q^{k-1}}^{2^{k-1}}, \end{array}$$

where the first line comes from *Proposition* 27. The second line comes from the fact that $\partial_{\vec{a}}\rho ={\langle \psi |w(\vec{a})\rangle }^{*}|\psi \rangle w(\vec{a})\rho$ for pure state ρ=|ψ〉〈ψ|. The third line comes from the fact that $\psi w{\left(\vec{a}\right)}^{*}\psi =d^{n}\;\Xi_{\rho }\left(\vec{a}\right)$, and the forth line comes Eq. **32** in *Proposition* 28. By using the inductive relation ${\left\|\rho \right\|}_{Q^{k}}^{2^{k}}=d^{n(2^{k-1}-2)}{\left\|{\text{Ξ}}_{\rho }\right\|}_{l^{2^{k-1}}}^{2^{k-1}}{\left\|\rho \right\|}_{Q^{k-1}}^{2^{k-1}}$, we get the result.

Remark 48: An n-qudit unit vector, $|\psi \rangle =\sum_{\vec{x}}f_{\psi }(\vec{x})|\vec{x}\rangle$ in the computational basis, gives the pure state $\rho_{\psi }=\left|\psi \rangle \langle \psi \right|=\sum_{\vec{a}}\Xi_{\rho_{\psi }}\left(\vec{a}\right)w\left(\vec{a}\right)$. In case $\rho_{\psi }$ is diagonal in the computational basis, i.e., $\rho_{\psi }=|\vec{x}\rangle \langle \vec{x}|$, we infer from *Propositions* 29 and 47 that ${\left\|\rho_{\psi }\right\|}_{Q^{k}}={\left\|f_{\psi }\right\|}_{U^{k}}=d^{-n\frac{k+1}{2^{k}}}$. But generally ${\left\|\rho_{\psi }\right\|}_{Q^{k}}\neq {\left\|f_{\psi }\right\|}_{U^{k}}$. In the simplest case k=1, using *Proposition* 2, ${\left\|\rho_{\psi }\right\|}_{Q^{1}}=\frac{1}{d^{n}}$, while ${\left\|f_{\psi }\right\|}_{U^{1}}=|E_{\vec{x}}f_{\psi }(\vec{x})|$, which depends on the vector |ψ〉.

Next we show logarithmic convexity of the quantum uniformity measures in the special case of pure states and certain k. It would be interesting to generalize this to mixed states and arbitrary k.

Proposition 49 (Log-convexity of the quantum uniformity norm). *Let* ρ *be a pure state in* $L(H^{⊗n})$ *for integer local dimension* d⩾2. *Then*

[56] $$\begin{array}{c} {\left\|\rho \right\|}_{Q^{2}}⩽{\left\|\rho \right\|}_{Q^{1}}^{1/2}{\left\|\rho \right\|}_{Q^{3}}^{1/2}. \end{array}$$

*Moreover, equality holds in Eq. **56**, if and only if* ρ *is a stabilizer state*.

***Proof:*** From *Proposition* 39, we infer that ${\left\|\rho \right\|}_{Q^{2}}={\left\|{\text{Ξ}}_{\rho }\right\|}_{4}$. And from *Proposition* 19 and $\mathrm{Tr}\left\{\rho \right\}=1$ we infer that ${\left\|\rho \right\|}_{Q^{1}}^{2}=\frac{1}{d^{2n}}$. Therefore Eq. **56** is equivalent to

[57] $$\begin{array}{c} d^{n}{\left\|{\text{Ξ}}_{\rho }\right\|}_{l^{4}}^{2}⩽{\left\|\rho \right\|}_{Q^{3}}\;. \end{array}$$

Inserting the value of ${\left\|\rho \right\|}_{Q^{3}}$ found in *Proposition* 47, we find that one needs to verify that

[58] $$\begin{array}{c} d^{n}{\left\|{\text{Ξ}}_{\rho }\right\|}_{l^{4}}^{2}⩽d^{n/4}{\left\|{\text{Ξ}}_{\rho }\right\|}_{l^{4}}\;,\;\;\text{or}\;{\left\|{\text{Ξ}}_{\rho }\right\|}_{4}⩽d^{-3n/4}\;. \end{array}$$

Since $\Xi_{\rho }\left(\vec{a}\right)=\langle w(\vec{a}),\rho \rangle =\frac{1}{d^{n}}\mathrm{Tr}\left\{w{\left(\vec{a}\right)}^{*}\rho \right\}$, one infers from $\left|\mathrm{Tr}\left\{w(\vec{a})\rho \right\}\right|⩽\mathrm{Tr}\left\{\rho \right\}=1$,

$$\left|\Xi_{\rho }\left(\vec{a}\right)\right|⩽\frac{1}{d^{n}}\;.$$

Also ρ is pure, so $\mathrm{Tr}\left\{\rho^{2}\right\}=1$, and $\sum_{\vec{a}\in V^{n}}{\left|\Xi_{\rho }\left(\vec{a}\right)\right|}^{2}=\frac{1}{d^{n}}$. Then

[59] $$\begin{array}{c} \sum_{\vec{a}}{\left|\Xi_{\rho }\left(\vec{a}\right)\right|}^{4}⩽\max_{\vec{b}}{\left|\Xi_{\rho }\left(\vec{b}\right)\right|}^{2}\sum_{\vec{a}}{\left|\Xi_{\rho }\left(\vec{a}\right)\right|}^{2}⩽\frac{1}{d^{2n}}\frac{1}{d^{n}}\;, \end{array}$$

so Eq. **58** holds.

Equality pertains if and only if $|\Xi_{\rho }\left(\vec{a}\right)|\in \left\{0,\frac{1}{d^{n}}\right\}$ for every $\vec{a}$, which means the pure state ρ is a stabilizer state. This characterization of stabilizers follows from *Proposition* 14.

## 10. Characterization by Quantum Convolution

In this section, we give an alternative way to characterize the quantum uniformity measures using the Hadamard convolution ${⊠}_{H}$, previously introduced by the authors in refs. 31, 32, and 49. In the following, we define convolution by starting from the tensor product of 2 copies of the n-qudit Hilbert space $H^{⊗n}$, which we denote as $H_{I}$ and $H_{\mathrm{II}}$. In considering $H_{I}⊗H_{\mathrm{II}}$, we denote ${\mathrm{Tr}}_{\mathrm{II}}\left\{\;\cdot \;\right\}$ as the partial trace over $H_{\mathrm{II}}$, bringing one back to the Hilbert space $H_{I}=H^{⊗n}$.

Definition 50 (Hadamard Convolution): The Hadamard convolution of two n-qudit states ρ and σ is the state

[60] $$\begin{array}{c} \rho {⊠}_{H}\sigma ={\mathrm{Tr}}_{\mathrm{II}}\left\{V_{H}\left(\rho ⊗\sigma \right)V_{H}^{*}\right\}\;. \end{array}$$

In this expression, we choose $V_{H}=U_{H}^{⊗n}:=U_{H}^{\left(1,n+1\right)}⊗U_{H}^{\left(2,n+2\right)}⊗...⊗U_{H}^{\left(n,2n\right)}$. Here $U_{H}$ is the 2-qudit Hadamard unitary

[61] $$\begin{array}{c} U_{H}=\sum_{x,y\in {\mathbb{Z}}_{d}}|x\rangle \langle x+y|⊗|y\rangle \langle x-y|, \end{array}$$

and $U_{H}^{\left(i,n+j\right)}$ denotes the action of $U_{H}$ on the ith qudit in $H_{I}$ and the jth qudit in $H_{\mathrm{II}}$. The corresponding convolutional channel $E_{H}$ is

[62] $$\begin{array}{c} E_{H}\left(\;\cdot \;\right)={\mathrm{Tr}}_{\mathrm{II}}\left\{V_{H}\;\cdot \;V_{H}^{*}\right\}\;. \end{array}$$

Lemma 51. *Let* $B=\sum_{\vec{a}}\Xi \left(\vec{a}\right)w\left(\vec{a}\right)$ *be an* n*-qudit transformation of local dimension* d. *Then*

[63] $$\begin{array}{c} {\left\|B{⊠}_{H}B\right\|}_{2}^{1/2}=d^{n/2}{\left(\sum_{\vec{a}}{\left|{\text{Ξ}}_{B}(\vec{a})\right|}^{4}\right)}^{1/4}=d^{n/2}{\left\|{\text{Ξ}}_{B}\right\|}_{4}\;. \end{array}$$

***Proof:*** This is a direct consequence of proposition 75 in ref. 32, noting that Ξ is normalized differently in that work. In more detail, the Hadamard convolution of two Weyl operators satisfies:

[64] $$\begin{array}{c} w\left(\vec{a}\right){⊠}_{H}w\left(\vec{b}\right)=d^{n}\delta_{\vec{a},\vec{b}}\;\omega^{-\langle {\vec{a}}_{1}{\vec{a}}_{2}\rangle }Z^{{\vec{a}}_{1}}w\left(\vec{a}\right)\;. \end{array}$$

Thus $B{⊠}_{H}B=\sum_{\vec{a}}d^{n}\Xi_{B}{\left(\vec{a}\right)}^{2}w^{\prime }\left(\vec{a}\right)$, where $w^{\prime }\left(\vec{a}\right)=Z^{{\vec{a}}_{1}}w\left(\vec{a}\right)$, up to a phase, is an orthonormal basis in the inner product determined by the Schatten 2-norm. In fact Eq. **34** shows the Schatten 2-norm squared of B equals the $\ell^{2}$ norm of $\Xi_{B{⊠}_{H}B}$. This establishes the proposition as claimed.

Proposition 52. *For* $B\in L(H^{⊗n})$, *we have both*

$$\begin{array}{cc} & d^{n/2}{\left\|B\right\|}_{Q^{2}}={\left\|B{⊠}_{H}B\right\|}_{2}^{1/2}\;,\\ & d^{n/2}{\left\|B\right\|}_{Q^{k+1}}^{2^{k+1}}\\ & =\underset{{\vec{a}}_{1},...,{\vec{a}}_{k-1}}{E}{\left\|\left(\partial_{{\vec{a}}_{k-1},{\vec{a}}_{k-2},...,{\vec{a}}_{1}}B){⊠}_{H}(\partial_{{\vec{a}}_{k-1},{\vec{a}}_{k-2},...,{\vec{a}}_{1}}B\right)\right\|}_{2}^{2}\;. \end{array}$$

***Proof:*** In *Proposition* 39, we have shown that ${\left\|B\right\|}_{Q^{2}}={\left\|{\text{Ξ}}_{B}\right\|}_{l^{4}}$. In *Proposition* 51, we show that ${\left\|B{⊠}_{H}B\right\|}_{2}^{1/2}=d^{n/2}{\left\|{\text{Ξ}}_{B}\right\|}_{l^{4}}$. In addition, by *Proposition* 27, one infers ${\left\|B\right\|}_{Q^{k+1}}^{2^{k+1}}$ satisfies

[65] $$\begin{array}{c} {\left\|B\right\|}_{Q^{k+1}}^{2^{k+1}}=\underset{{\vec{a}}_{1},...,{\vec{a}}_{k-1}}{E}{\left\|\partial_{{\vec{a}}_{k-1},{\vec{a}}_{k-2},...,{\vec{a}}_{1}}B\right\|}_{Q^{2}}^{4}\;, \end{array}$$

as claimed.

## 11. Overlap with the Clifford Hierarchy

We have shown that the measures ${\left\|\cdot \right\|}_{Q^{k+1}}$ characterize exactly whether a unitary U is an element of the kth-level $C^{\left(k\right)}$ of the Clifford hierarchy. Here we introduce a different measure ${\left\|U\right\|}_{q^{k+1}}$, which we use to bound the $L^{2}$-Schatten distance between a given unitary U and $C^{\left(k\right)}$. In classical HOFA the analogs of such measures have been extensively studied.

Definition 53: The overlap of a unitary U with the kth level $C^{\left(k\right)}$ of the Clifford hierarchy is

[66] $$\begin{array}{c} {\left\|U\right\|}_{q^{k+1}}=\max_{V\in C^{\left(k\right)}}{\left|\left\langle V,U\right\rangle \right|}^{2}. \end{array}$$

As $C^{\left(k\right)}\subset C^{\left(k+1\right)}$, then we have ${\left\|U\right\|}_{q^{k}}⩽{\left\|U\right\|}_{q^{k+1}}$ for any k.

Proposition 54. *Given an* n*-qudit unitary* U *and* k⩾1, *one has*

[67] $$\begin{array}{c} {\left\|U\right\|}_{q^{k}}=1\;\;\mathrm{iff}\;{\left\|U\right\|}_{Q^{k}}=1\;. \end{array}$$

*In other words*, U *is in the* kth *level of Clifford hierarchy iff* $U_{Q^{k+1}}=1$

***Proof:*** By the inductive definition, we only need to prove the case where k=1. That, U is equal to identity up to some phase, iff ${\left\|U\right\|}_{Q^{1}}=1$. This is because ${\left\|U\right\|}_{Q^{1}}^{2}=|\frac{1}{d^{n}}\text{Tr}\left\{U\right\}{|}^{2}=1$ iff U is equal to identity up to some phase.

Lemma 55. *For any two* n*-qudit unitaries* U,V,

[68] $$\begin{array}{c} E_{\vec{a}}{\left|\langle \partial_{\vec{a}}U,\partial_{\vec{a}}V\rangle \right|}^{2}=\sum_{\vec{a}}{\left|\Xi_{U^{*}V}\left(\vec{a}\right)\right|}^{4}\;. \end{array}$$

***Proof:*** This is a consequence of Eq. **20** and *Proposition* 39, with $B=U^{*}V$.

Theorem 56 (Overlap and the Uniformity Measures). *Given an* n*-qudit unitary* U, *and* k⩾1,

[69] $$\begin{array}{c} {\left\|U\right\|}_{q^{k}}⩽{\left\|U\right\|}_{Q^{k}}\;. \end{array}$$

***Proof:*** First, let us prove that for any unitaries U and V,

[70] $$\begin{array}{c} E_{\vec{a}}|\left\langle \partial_{\vec{a}}U,\partial_{\vec{a}}V\right\rangle {|}^{2}⩾{\left|\left\langle U,V\right\rangle \right|}^{4}\;. \end{array}$$

In fact by *Lemma* 55 and $\langle \partial_{\vec{a}}U,\partial_{\vec{a}}V\rangle =\frac{1}{d^{n}}\mathrm{Tr}\left\{\partial_{\vec{a}}\left(U^{*}V\right)\right\}$,

$$\begin{array}{cc} E_{\vec{a}}{\left|\left\langle \partial_{\vec{a}}U,\partial_{\vec{a}}V\right\rangle \right|}^{2}= & \sum_{\vec{a}}{\left|\Xi_{U^{*}V}\left(\vec{a}\right)\right|}^{4}⩾{\left|\Xi_{U^{*}V}\left(0\right)\right|}^{4}\\ = & {\left|\frac{1}{d^{n}}\mathrm{Tr}\left\{U^{*}V\right\}\right|}^{4}={\left|\langle U,V\rangle \right|}^{4}\;, \end{array}$$

as claimed in Eq. **68**.

The definition of ${\left\|U\right\|}_{q^{k+1}}$, ensures that there exists a unitary $V\in C^{\left(k\right)}$, such that

[71] $$\begin{array}{c} {\left\|U\right\|}_{q^{k+1}}={\left|\left\langle U,V\right\rangle \right|}^{2}\;. \end{array}$$

Also *Theorem* 5 shows that $U\in C^{k}$ means that $\partial_{{\vec{a}}_{k},...,{\vec{a}}_{1}}V$ is equal to I, up to a phase. Thus *Proposition* 2 lets us write

$$\begin{array}{cc} {\left\|U\right\|}_{Q^{k+1}}^{2^{k+1}} & =E_{{\vec{a}}_{1},...,{\vec{a}}_{k}}{\left|\left\langle \partial_{{\vec{a}}_{k},...,{\vec{a}}_{1}}U,\partial_{{\vec{a}}_{k},...,{\vec{a}}_{1}}V\right\rangle \right|}^{2}\\ & ⩾E_{{\vec{a}}_{1},...,{\vec{a}}_{k-1}}{\left|\left\langle \partial_{{\vec{a}}_{k-1},...,{\vec{a}}_{1}}U,\partial_{{\vec{a}}_{k-1},...,{\vec{a}}_{1}}V\right\rangle \right|}^{4}\\ & ⩾(E_{{\vec{a}}_{1},...,{\vec{a}}_{k-1}}|\left\langle \partial_{{\vec{a}}_{k-1},...,{\vec{a}}_{1}}U,\partial_{{\vec{a}}_{k-1},...,{\vec{a}}_{1}}V\right\rangle {{|}^{2})}^{2}\\ & ⩾...⩾{\left({\left|\langle U,V\rangle \right|}^{2}\right)}^{2^{k}}. \end{array}$$

In the second inequality, we use the Schwarz inequality. Iterating this procedure establishes the desired bound.

## 12. q-HOFA Gives a Clifford Hierarchy Test

We consider an important task in quantum property testing, called Clifford hierarchy testing. Given a unitary U and an integer k, the goal is to determine whether U belongs to the k-Clifford hierarchy $C^{\left(k\right)}$, or if it is ϵ-far from $C^{\left(k\right)}$. This task can be regarded as the quantum counterpart of low-degree testing of polynomials in classical theory.

Clifford testing and magic entropy have been proposed by the authors using their quantum convolution (49). Let us first consider the n-qudit system with d being odd prime. The Hadamard convolution is given in *Definition* 50. We can generalize the test to any k-Clifford hierarchy, using convolution-swap testing.

Box 1. kth Clifford-Hierarchy Testing for odd prime d

1. Chose ${\vec{a}}_{1},...,{\vec{a}}_{k-2}$ from $V^{n}$ independently and uniformly and random.
2. Prepare 4 copies of the Choi states $J_{\partial_{{\vec{a}}_{1},...,{\vec{a}}_{k-2}}U}$ for the unitary $\partial_{{\vec{a}}_{1},...,{\vec{a}}_{k-2}}U$, and apply the convolution for each 2 copies of $J_{\partial_{{\vec{a}}_{1},...,{\vec{a}}_{k-2}}U}$, and get 2 copies of $J_{\partial_{{\vec{a}}_{1},...,{\vec{a}}_{k-2}}U}{⊠}_{H}J_{\partial_{{\vec{a}}_{1},...,{\vec{a}}_{k-2}}U}$;
3. Perform the swap test for the 2 copies of $J_{\partial_{{\vec{a}}_{1},...,{\vec{a}}_{k-2}}U}{⊠}_{H}J_{\partial_{{\vec{a}}_{1},...,{\vec{a}}_{k-2}}U}$. If the output is 0, it passes the test; otherwise, it fails.

Theorem 57. *The probability of acceptance for the above* k*th-Clifford hierarchy testing can be written in terms of the quantum uniformity norm:*

[72] $$\begin{array}{c} P_{k}\left[U\right]=\frac{1}{2}\left[1+{\left\|U\right\|}_{Q^{k+1}}^{2^{k}}\right]. \end{array}$$

***Proof:*** Based on the protocol for the kth-Clifford hierarchy testing, the probability can be written as

$$\begin{array}{c} P_{k}\left[U\right]=\frac{1}{2}\left[1+\underset{{\vec{a}}_{1},...,{\vec{a}}_{k-2}}{E}\mathrm{Tr}\left\{{\left(J_{\partial_{{\vec{a}}_{1},...,{\vec{a}}_{k-2}}U}{⊠}_{H}J_{\partial_{{\vec{a}}_{1},...,{\vec{a}}_{k-2}}U}\right)}^{2}\right\}\right]. \end{array}$$

Then using *Proposition* 52, we obtain the result.

We can use other quantum convolutions, in addition to the Hadamard convolution, to implement the Clifford hierarchy testing. In the n-qubit case, we need to change the definition of quantum convolution.

Definition 58: The convolution of three n-qubit states $\rho_{1},\rho_{2},\rho_{3}$ is

[73] $$\begin{array}{c} {⊠}_{3}\left(\rho_{1},\rho_{2},\rho_{3}\right)={\mathrm{Tr}}_{1^{c}}\left\{V\rho_{1}⊗\rho_{2}⊗\rho_{3}V^{*}\right\}, \end{array}$$

where $V=U^{⊗n}=U^{\left(1,n+1,2n+1\right)}⊗U^{\left(2,n+2,2n+2\right)}⊗....⊗U^{\left(n,2n,3n\right)}$, and U is a K-qubit unitary constructed using CNOT gates:

[74] $$\begin{array}{c} U:=\left(\prod_{j=1}^{3}CNOT_{j\to 1}\right)\left(\prod_{i=1}^{3}CNOT_{1\to i}\right), \end{array}$$

and $CNOT_{2\to 1}|x\rangle |y\rangle =|x+y\rangle |y\rangle$ for any $x,y\in F_{2}$.

This gives rise to a Clifford hierarchy test for qubits. The probability of acceptance for the above kth-Clifford hierarchy testing is

[75] $$\begin{array}{c} P_{k}\left[U\right]=\frac{1}{2}\left[1+E_{{\vec{a}}_{1},...,{\vec{a}}_{k-2}\in V^{n}}\mathrm{Tr}\left\{{\left({⊠}_{3}J_{\partial_{{\vec{a}}_{1},...,{\vec{a}}_{k-2}U}}\right)}^{2}\right\}\right]. \end{array}$$

Corollary 59. *The success probability of the* k*-th level of Clifford hierarchy testing is lower bounded by the maximal overlap with the* k*-th Clifford hierarchy*,

[76] $$\begin{array}{c} P_{k}\left(U\right)⩾\frac{1}{2}\left[1+{\left\|U\right\|}_{q^{k+1}}^{2^{k+1}}\right]. \end{array}$$

## 13. Outlook

In this work, we propose a framework of quantum higher-order Fourier analysis and show its application in quantum computation. There are still many interesting open problems. The inverse quantum uniformity norm conjecture can be regarded as a quantum generalization of the higher-order Goldreich-Levin algorithm. This conjecture is if $U_{Q^{3}}⩾c$, then there exists a Clifford unitary V such that the overlap between U and V is bounded below by some constant, independent of the number of qudits. If this conjecture is true, it is natural to ask: Can one find an algorithm that makes polynomially many queries of a given unitary U and produces a decomposition of U as a sum of Clifford unitaries and a small error term? In general, if ${\left\|U\right\|}_{Q^{k+1}}⩾c$, does there exist a unitary V in kth-level of Clifford hierarchy, such that the overlap between U and V is bounded below by some constant, which is independent of the number of qudits?

Quantum teleportation is a crucial element that allows universal fault-tolerant quantum computation through stabilizer codes (2). An important problem is to find the depth of teleportation to implement quantum gates in the Clifford hierarchy, where the depth of teleportation is a measure of the complexity of the gate to characterize the number of teleportation steps to implement a given gate in fault-tolerant quantum computation (50). Is there a connection between the quantum uniformity measures and teleportation depth?

## Supplementary Material

Appendix 01 (PDF)

## Data Availability

There are no data underlying this work.
