# Supplementary material for: Quantum higher-order Fourier analysis and the Clifford hierarchy
Source: Proc Natl Acad Sci U S A. 2025 Nov 7;122(45):e2515667122. doi: 10.1073/pnas.2515667122 (PMC12625977; doi:10.1073/pnas.2515667122)
Supplement: Supplementary file 1 — Appendix 01 (PDF) [file pnas.2515667122.sapp.pdf]

Supporting Information Text

Here we derive some assertions in the main text that require extra space. The propositions are numbered here with reference to the corresponding statements in the main text.

A. Generalized Quantum Schwarz Inequality

Proposition 41. *For $k=3$,*

$$|\langle\{\,B_{\vec{u}}\,\}_{\vec{u}\in\{\,0,1\,\}^k}\rangle_{Q^k}| \leqslant \Pi_{\vec{u}\in\{\,0,1\,\}^k}\, ||B_{\vec{u}}||_{Q^k} \ . \qquad [A.1]$$

In particular,

$$\begin{aligned}&\left|\langle B_{000},B_{001},B_{010},B_{011},B_{100},B_{101},B_{110},B_{111}\rangle_{Q^3}\right|\\ &\quad\leqslant ||B_{000}||_{Q^3}\ ||B_{001}||\ ||B_{010}||_{Q^3}\ ||B_{011}||_{Q^3}\ ||B_{100}||_{Q^3}\ ||B_{101}||_{Q^3}\ ||B_{110}||_{Q^3}\ ||B_{111}||_{Q^3} \ .\end{aligned}$$

Proof. Start by using the Schwarz inequality of Proposition 36 to give

$$\begin{aligned}&\left|\langle B_{000},B_{001},B_{010},B_{011},B_{100},B_{101},B_{110},B_{111}\rangle_{Q^3}\right|\\ &\quad\leqslant \left|\langle B_{000},B_{001},B_{010},B_{011},B_{000},B_{001},B_{010},B_{011}\rangle_{Q^3}\right|^{1/2}\ \left|\langle B_{100},B_{101},B_{110},B_{111},B_{100},B_{101},B_{110},B_{111}\rangle_{Q^3}\right|^{1/2} . \qquad [A.2]\end{aligned}$$

Now one can apply the second identity in Lemma 39, followed by the Schwarz inequality of Proposition 36 which shows that the first term in Eq. (A.2) satisfies

$$\begin{aligned}&\left|\langle B_{000},B_{001},B_{010},B_{011},B_{000},B_{001},B_{010},B_{011}\rangle_{Q^3}\right|^{1/2} = \left|\langle B_{011},B_{010},B_{011},B_{010},B_{001},B_{000},B_{001},B_{000}\rangle_{Q^3}\right|^{1/2}\\ &\quad\leqslant \left|\langle B_{011},B_{010},B_{011},B_{010},B_{011},B_{010},B_{011},B_{010}\rangle_{Q^3}\right|^{1/4}\left|\langle B_{001},B_{000},B_{001},B_{000},B_{001},B_{000},B_{001},B_{000}\rangle_{Q^3}\right|^{1/4} \ . \qquad [A.3]\end{aligned}$$

One can continue this process. For the first term on the right of Eq. (A.3),

$$\begin{aligned}&\left|\langle B_{011},B_{010},B_{011},B_{010},B_{011},B_{010},B_{011},B_{010}\rangle_{Q^3}\right|^{1/4} = \left|\langle B^*_{011},B^*_{011},B^*_{010},B^*_{010},B^*_{011},B^*_{011},B^*_{010},B^*_{010}\rangle_{Q^3}\right|^{1/4}\\ &\quad= \left|\langle B^*_{011},B^*_{011},B^*_{011},B^*_{011},B^*_{010},B^*_{010},B^*_{010},B^*_{010}\rangle_{Q^3}\right|^{1/4}\\ &\quad\leqslant \left|\langle B^*_{011},B^*_{011},B^*_{011},B^*_{011},B^*_{011},B^*_{011},B^*_{011},B^*_{011}\rangle_{Q^3}\right|^{1/8}\ \left|\langle B^*_{010},B^*_{010},B^*_{010},B^*_{010},B^*_{010},B^*_{010},B^*_{010},B^*_{010}\rangle_{Q^3}\right|^{1/8}\\ &\quad= ||B_{011}||_{Q^3}\ ||B_{010}||_{Q^3} \ . \qquad [A.4]\end{aligned}$$

Here the first equality comes from the first identity in Lemma 39, while the second equality comes from the second identity. One obtains the final inequality by using the quantum Schwarz inequality in Proposition 36.

The second term on the right of Eq. (A.3) satisfies a similar bound, so that

$$\left|\langle B_{001},B_{000},B_{001},B_{000},B_{001},B_{000},B_{001},B_{000}\rangle_{Q^3}\right|^{1/4} \leqslant ||B_{000}||_{Q^3}\ ||B_{001}||_{Q^3} \ . \qquad [A.5]$$

Inserting Eq. (A.4) and Eq. (A.5) into Eq. (A.3) gives

$$\left|\langle B_{000},B_{001},B_{010},B_{011},B_{000},B_{001},B_{010},B_{011}\rangle_{Q^3}\right|^{1/2} \leqslant ||B_{000}||_{Q^3}\ ||B_{001}||_{Q^3}\ ||B_{010}||_{Q^3}\ ||B_{011}||_{Q^3} \ . \qquad [A.6]$$

The last term in Eq. (A.2) is similar to the term we just bounded, except it is a bound with $B_{1\vec{u}_2}$ replacing $B_{0\vec{u}_2}$. Making this substitution, one has

$$\left|\langle B_{100},B_{101},B_{110},B_{111},B_{100},B_{101},B_{110},B_{111}\rangle_{Q^3}\right|^{1/2} \leqslant ||B_{100}||_{Q^3}\ ||B_{101}||_{Q^3}\ ||B_{110}||_{Q^3}\ ||B_{111}||_{Q^3} \ . \qquad [A.7]$$

Inserting the bounds Eq. (A.6) and Eq. (A.7) into Eq. (A.2) yields

$$\left|\langle B_{000},B_{001},B_{010},B_{011},B_{100},B_{101},B_{110},B_{111}\rangle_{Q^3}\right| \leqslant \langle\{\,B_{\vec{u}}\,\}_{\vec{u}\in\{\,0,1\,\}^3}\rangle_{Q^3} \leqslant \Pi_{\vec{u}\in\{\,0,1\,\}^3}\, ||B_{\vec{u}}||_{Q^3} \ ,$$

and completes the proof. □

Proposition 45. *Given two operators $A,B\in L(\mathcal{H}^{\otimes n})$ and an integer $k\geqslant 1$, one has for Schatten norms $||\cdot||_r$ that*

$$(\mathbb{E}_{\vec{a}\in V^n}\|A(\vec{a})B\|_r^s)^{1/s} \leqslant \|A\|_p\|B\|_q, \qquad [A.8]$$

where

$$p=q=\frac{2^k}{k+1}, \quad r=\frac{2^{k-1}}{k}, \quad s=2^{k-1}.$$

Proof. We use Hölder inequality for A_1, A_2, A_3, namely

$$\|A_1 A_2 A_3\|_r \leqslant \|A_1\|_{p_1} \|A_2\|_{p_2} \|A_3\|_{p_3} \ , \qquad \text{[A.9]}$$

where $\frac{1}{r} = \frac{1}{p_1} + \frac{1}{p_2} + \frac{1}{p_3}$. We take $p_1 = \frac{sp}{s-p}$, $p_2 = s$, and $p_3 = \frac{sq}{s-q}$. We use the identity

$$|A(\vec{a})B|^r = \left(|A(\vec{a})B|^2\right)^{r/2} = \left(B^* \left(A^* A\right)(\vec{a}) B\right)^{r/2} \ .$$

Suppose $B = |B|\, V$ for unitary V. Then one has for any $0 < r$ that $\mathrm{Tr}\left\{|A(\vec{a})B|^r\right\} = \mathrm{Tr}\left\{\left(|B|\,|A|^2 (\vec{a})\,|B|\right)^{r/2}\right\} = \mathrm{Tr}\left\{\left(|A|\,(\vec{a})\,|B|\right)^r\right\}$. Hence,

$$\begin{aligned}
\left(\frac{1}{d^n}\mathrm{Tr}\left\{|A(\vec{a})B|^r\right\}\right)^{\frac{1}{r}} &= \left(\frac{1}{d^n}\mathrm{Tr}\left\{\left(|A|\,(\vec{a})\,|B|\right)^r\right\}\right)^{\frac{1}{r}} = \left(\frac{1}{d^n}\mathrm{Tr}\left\{\left(|A|^{\frac{s-p}{s}}\,(\vec{a})\,|A|^{\frac{p}{s}}\,(\vec{a})\,|B|^{\frac{q}{s}}\,|B|^{\frac{s-q}{s}}\right)^r\right\}\right)^{\frac{1}{r}} \\
&\leqslant \left(\frac{1}{d^n}\mathrm{Tr}\left\{(|A|^{\frac{s-p}{s}})^{\frac{sp}{s-p}}\right\}\right)^{\frac{s-p}{sp}} \left(\frac{1}{d^n}\mathrm{Tr}\left\{\left||A|^{\frac{p}{s}}(\vec{a})|B|^{\frac{q}{s}}\right|^s\right\}\right)^{\frac{1}{s}} \left(\frac{1}{d^n}\mathrm{Tr}\left\{(|B|^{\frac{s-q}{s}})^{\frac{sq}{s-q}}\right\}\right)^{\frac{s-q}{sq}} \ .
\end{aligned}$$

In the last inequality we use Eq. (A.9). By rewriting it, we have

$$\|A(\vec{a})B\|_r^s \leqslant \|A\|_p^{s-p} \|B\|_q^{s-q} \frac{1}{d^n}\mathrm{Tr}\left\{\left|w(\vec{a})|A|^{\frac{p}{s}} w(\vec{a})^* |B|^{\frac{q}{s}}\right|^s\right\} \ . \qquad \text{[A.10]}$$

If $k = 1$,

$$\mathbb{E}_{\vec{a}\in V^n} \frac{1}{d^n}\mathrm{Tr}\left\{\left|w(\vec{a})|A|^{\frac{p}{s}} w(\vec{a})^* |B|^{\frac{q}{s}}\right|^s\right\} = \mathbb{E}_{\vec{a}\in V^n} \frac{1}{d^n}\mathrm{Tr}\left\{w(\vec{a})|A|w(\vec{a})^*|B|\right\} = \|A\|_p^p \|B\|_q^q \ .$$

When $k \geqslant 2$,

$$\begin{aligned}
\mathbb{E}_{\vec{a}\in V^n} \frac{1}{d^n}\mathrm{Tr}\left\{\left|w(\vec{a})|A|^{\frac{p}{s}} w(\vec{a})^* |B|^{\frac{q}{s}}\right|^s\right\} =& \mathbb{E}_{\vec{a}\in V^n} \frac{1}{d^n}\mathrm{Tr}\left\{\left(w(\vec{x})|A|^{\frac{p}{s}} w(\vec{a})^* |B|^{\frac{q}{s}} |B|^{\frac{q}{s}} w(\vec{a})|A|^{\frac{p}{s}} w(\vec{a})^*\right)^{\frac{s}{2}}\right\} \\
=& \mathbb{E}_{\vec{a}\in V^n} \frac{1}{d^n}\mathrm{Tr}\left\{\left(w(\vec{a})|A|^{\frac{2p}{s}} w(\vec{a})^* |B|^{\frac{2q}{s}}\right)^{\frac{s}{2}}\right\} \\
\leqslant& \||A|^{\frac{2p}{s}}\|_{s/2}^{s/2} \, \||B|^{\frac{2q}{s}}\|_{s/2}^{s/2} \\
=& \|A\|_p^p \|B\|_q^q \ .
\end{aligned}$$

Here the inequality comes from the Lemma 43. Combined with Eq. (A.10), we get

$$\mathbb{E}_{\vec{a}\in V^n} \|A(\vec{a})B\|_r^s \leqslant \|A\|_p^s \|B\|_q^s \ ,$$

which completes the proof. □

B. Calculation of the Quantum Measures for Gates in §7 of the Main Text

We give the detailed calculations of the quantum uniformity measures for several examples of quantum gates, most of which are stated in §7.

Example 1 (The Weyl Gate). For $b = (p, q)$, the one-qudit Weyl (or Pauli) gates are $w(a) = \zeta^{-pq} Z^p X^q$. The n-qudit Weyl gates are tensor products of 1-qudit gates, so their quantum uniformity measures are the nth power of the one-qudit measures. We claim that

$$\left|\left|w(\vec{b})\right|\right|_{Q^1} = \delta_{\vec{b},\vec{0}} \ , \quad \text{and} \quad \left|\left|w(\vec{b})\right|\right|_{Q^k} = 1, \quad \forall k \geqslant 2. \qquad \text{[B.11]}$$

To calculate the $k = 1$ norm, use Proposition 25. In the computational basis, Z is diagonal and X is off-diagonal. So $\mathrm{Tr}\left\{Z^p X^q\right\} = \mathrm{Tr}\left\{Z^p\right\}\delta_{q,0} = d\delta_{q,0}$. Thus,

$$\left|\left|w(\vec{b})\right|\right|_{Q^1} = \left|\frac{1}{d^n}\mathrm{Tr}\left\{w(\vec{b})\right\}\right| = \delta_{\vec{b},\vec{0}} \ .$$

To calculate the $k = 2$ norm, observe that

$$\begin{aligned}
\left|\left|w(\vec{b})\right|\right|_{Q^2}^4 =& \mathbb{E}_{\vec{a}} \left|\left|\partial_{\vec{a}} w(\vec{b})\right|\right|_{Q_1}^2 = \mathbb{E}_{\vec{a}} \left|\frac{1}{d^n}\mathrm{Tr}\left\{\partial_{\vec{a}} w(\vec{b})\right\}\right|^2 \\
=& \mathbb{E}_{\vec{a}} \left|\frac{1}{d^n}\mathrm{Tr}\left\{w(\vec{a})w(\vec{b})w(\vec{a})^* w(\vec{b})^*\right\}\right|^2 = \mathbb{E}_{\vec{a}} \left|\frac{1}{d^n}\mathrm{Tr}\left\{I\right\}\right|^2 = 1.
\end{aligned}$$

Finally, $\left|\left|w(\vec{b})\right|\right|_{Q^k} = 1$ for $k \geqslant 3$, as $\left|\left|w(\vec{b})\right|\right|_{Q^k}^{2^k} = \mathbb{E}_{\vec{a}} \left|\left|\partial_{\vec{a}} w(\vec{b})\right|\right|_{Q_{k-1}}^{2^{k-1}} = \mathbb{E}_{\vec{a}} \left|\left|I\right|\right|_{Q^{k-1}}^{2^{k-1}} = 1.$

Example 2 (The Fourier Gate). The Fourier gate on an n-qudit system plays an important role in quantum computation,

$$F = \frac{1}{d^{n/2}} \sum_{\vec{x},\vec{y}} \omega^{\vec{x}\cdot\vec{y}} |\vec{x}\rangle \langle\vec{y}| \ , \quad where \quad \omega = e^{2\pi i/d} \ .$$

The quantum uniformity measures of F are

$$||F||_{Q^1} = \begin{cases} 0 \ , \ \text{if } d=2 \\ \frac{1}{d^n}, \ \text{if } d \text{ is odd prime} \end{cases} , \quad ||F||_{Q^2} = \begin{cases} \frac{1}{2^{n/4}}, & \text{if } \ d=2 \\ \frac{1}{d^{n/2}}, & \text{if d is odd prime} \end{cases} , \quad ||F||_{Q^k} = 1, \quad \forall k \geqslant 3 \ . \qquad \text{[B.12]}$$

In verifying Eq. (B.12) we use that the n-qudit F is the tensor product of 1-qudit transforms F_1. Thus $||F||_{Q^k} = ||F_1||_{Q^k}^n$.

The case $k=1$: By Proposition 25,

$$||F_1||_{Q^1} = \frac{1}{d} \left|\text{Tr}\left\{F_1\right\}\right| . \qquad \text{[B.13]}$$

The trace $\text{Tr}\{F_1\}$ can be evaluated in the computational basis as a Gauss sum:

$$\text{Tr}\left\{F_1\right\} = \frac{1}{\sqrt{d}} \sum_{x\in\mathbb{Z}_d} \omega^{x^2} = \begin{cases} 1+i, & \text{if } \ d\equiv 0 \mod 4 \\ 1, & \text{if } \ d\equiv 1 \mod 4 \\ 0, & \text{if } \ d\equiv 2 \mod 4 \\ i, & \text{if } \ d\equiv 3 \mod 4 \end{cases} , \quad \text{so} \quad \left|\text{Tr}\left\{F_1\right\}\right| = \begin{cases} 0 \ , & \text{if } \ d=2 \\ 1 \ , & \text{if } d \text{ is an odd prime} \end{cases} . \qquad \text{[B.14]}$$

Inserting this into Eq. (B.13) gives for the one-qudit system,

$$||F_1||_{Q^1} = \begin{cases} 0 \ , & \text{if } \ d=2 \\ \frac{1}{d} \ , & \text{if } d \text{ is an odd prime} \end{cases} , \quad \text{and for } n \text{ qudits} \quad ||F||_{Q^1} = \begin{cases} 0 \ , & \text{if } \ d=2 \\ \frac{1}{d^n} \ , & \text{if } d \text{ is an odd prime} \end{cases} .$$

The case $k=2$: Let $a=(p,q)\in\mathbb{Z}_d\times\mathbb{Z}_d$. We claim that $\partial_{\vec{a}}F_1$ is equal to $w(p-q,p+q)$, up to some phase. In fact,

$$\partial_a F_1 = w(a)F_1 w(a)^* F_1^* = Z^p X^q F_1 X^{-q} Z^{-p} F_1^* = Z^p X^q (F_1 X^{-q} F_1^*)(F_1 Z^{-p} F_1^*) = \omega^{q^2} Z^{p-q} X^{p+q} = \zeta^{p^2+q^2} w(p-q,p+q) \ .$$

Here we used the fact that $F_1 Z F_1^* = X^{-1}$, and $F_1 X F_1^* = Z$. Then $||F_1||_{Q^2}$ can be computed using the recursion relation in the main text, namely Proposition 2. This gives,

$$||F_1||_{Q^2}^4 = \mathbb{E}_a \, ||\partial_a F_1||_{Q^1}^2 = \frac{1}{d^2} \sum_{p,q\in\mathbb{Z}_d} ||w(p-q,p+q)||_{Q^1}^2 = \frac{1}{d^2} \sum_{p,q\in\mathbb{Z}_d} \delta_{p+q,0}\, \delta_{p-q,0} \ ,$$

where the last equality comes from Eq. (B.11). This is also equal to

$$||F_1||_{Q^2}^4 = \begin{cases} \frac{1}{2}, & \text{if } \ d=2 \\ \frac{1}{d^2}, & \text{if d is an odd prime} \end{cases} . \quad \text{Hence} \quad ||F||_{Q^2} = \begin{cases} \frac{1}{2^{n/4}}, & \text{if } \ d=2 \\ \frac{1}{d^{n/2}}, & \text{if d is an odd prime} \end{cases} . \qquad \text{[B.15]}$$

The case $k \geqslant 3$: Again one can use Proposition 2 to show

$$||F_1||_{Q^3}^8 = \mathbb{E}_a \, ||\partial_a F_1||_{Q^2}^4 = \frac{1}{d^2} \sum_{p,q\in\mathbb{Z}_d} ||w(p-q,p+q)||_{Q^2}^4 = 1 \ .$$

Hence $||F||_{Q^3} = ||F_1||_{Q^3} = 1$. The same argument shows that $||F||_{Q^k} = 1$ for any $k > 3$.

For $d=2$ case, namely for a qubit system, the Fourier gate F is known as Hadamard gate H. For $n=1$,

$$H_1 = \frac{1}{\sqrt{2}} \begin{bmatrix} 1 & 1 \\ 1 & -1 \end{bmatrix} . \qquad \text{[B.16]}$$

The corresponding quantum uniformity measures are

$$||H_1||_{Q^1} = 0 \ , \quad ||H_1||_{Q^2} = \frac{1}{2^{1/4}} \ , \quad ||H_1||_{Q^k} = 1 \ , \quad \forall k \geqslant 3 \ . \qquad \text{[B.17]}$$

Example 3 (The CNOT gate). Let us consider a two-qudit ($n=2$) CNOT gate

$$CNOT = \sum_{x,y\in\mathbb{Z}_d} |x\rangle\langle x| \otimes |x+y\rangle \langle y| \ .$$

This is an important gate for the generation of quantum entanglement. Here we compute the corresponding quantum uniformity norms

$$||CNOT||_{Q^1} = \frac{1}{d} \ , \quad ||CNOT||_{Q^2} = \frac{1}{\sqrt{d}} \ , \quad ||CNOT||_{Q^k} = 1 \ , \quad \forall k \geqslant 3. \qquad \text{[B.18]}$$

The case $k=1$: Using Proposition 2 or Proposition 25 in the main text for the case $n=2$, and the relation $\mathrm{Tr}\{CNOT\}=d$, we have

$$||CNOT||_{Q^1}=\frac{1}{d^2}|\mathrm{Tr}\{CNOT\}|=\frac{1}{d}\,.$$

The case $k=2$: It is convenient to know the action of CNOT on the Weyl operator $w(\vec{a})=w(a_1)\otimes w(a_2)$, where $\vec{a}=(a_1,a_2)$ and $a_j=(p_j,q_j)$. We claim that

$$CNOT\,w(\vec{a})\,CNOT^*=CNOT\,(w(p_1,q_1)\otimes w(p_2,q_2))\,CNOT^*=\zeta^{p_2(q_1+q_2)}w(p_1-p_2,q_1)\otimes w(p_2,q_1+q_2)\,. \tag{B.19}$$

As

$$w(p_1,q_1)\otimes w(p_2,q_2=w(\vec{a})=\zeta^{-p_1q_1+p_2q_2}\,(Z^{p_1}\otimes X^{q_2})\,(X^{q_1}\otimes Z^{p_2})\ , \tag{B.20}$$

one can use the actions

$$CNOT\,(Z^p\otimes X^q)\,CNOT^*=Z^p\otimes X^q\ ,\quad \text{and}\quad CNOT\,(X^q\otimes Z^p)\,CNOT^*=X^qZ^{-p}\otimes X^qZ^p \tag{B.21}$$

to obtain Eq. (B.19). The first identity in Eq. (B.21) comes from

$$\begin{aligned}CNOT\,(Z^p\otimes X^q)\,CNOT^*&=\sum_{a,b,c,d\in\mathbb{Z}_d}(|a\rangle\langle a|\otimes|a+b\rangle\langle b|)\ (Z^p\otimes X^q)\ (|c\rangle\langle c|\otimes|d\rangle\langle c+d|)\\&=\sum_{a,b,c,d\in\mathbb{Z}_d}\omega^{pc}\,(|a\rangle\langle a|\otimes|a+b\rangle\langle b|)\,(|c\rangle\langle c|\otimes|d+q\rangle\langle c+d|)\\&=\sum_{a,b,c,d\in\mathbb{Z}_d}\omega^{pc}\delta_{a,c}\delta_{b,d+q}\,|a\rangle\langle c|\otimes|a+b\rangle\langle c+d|\\&=\sum_{a,b\in\mathbb{Z}_d}\omega^{pa}\,|a\rangle\langle a|\otimes|a+b\rangle\langle a+b-q|=\sum_{a,b\in\mathbb{Z}_d}\omega^{pa}\,|a\rangle\langle a|\otimes|a+b+q\rangle\langle a+b|\\&=\sum_{a,b\in\mathbb{Z}_d}\omega^{pa}\,|a\rangle\langle a|\otimes|b+q\rangle\langle b|=Z^p\otimes X^q\,.\end{aligned}$$

The second identity in Eq. (B.21) is

$$\begin{aligned}CNOT\,(X^q\otimes Z^p)\,CNOT^*&=\sum_{a,b,c,d\in\mathbb{Z}_d}(|a\rangle\langle a|\otimes|a+b\rangle\langle b|)\ (X^q\otimes Z^p)\ (|c\rangle\langle c|\otimes|d\rangle\langle c+d|)\\&=\sum_{a,b,c,d\in\mathbb{Z}_d}\omega^{pd}\,(|a\rangle\langle a|\otimes|a+b\rangle\langle b|)\ (|c+q\rangle\langle c|\otimes|d\rangle\langle c+d|)\\&=\sum_{a,b,c,d\in\mathbb{Z}_d}\omega^{pd}\delta_{a,c+q}\delta_{b,d}\,|a\rangle\langle c|\otimes|a+b\rangle\langle c+d|\\&=\sum_{a,b\in\mathbb{Z}_d}\omega^{pb}\,|a\rangle\langle a-q|\otimes|a+b\rangle\langle a+b-q|=\sum_{a,b\in\mathbb{Z}_d}\omega^{pb}\,|a+q\rangle\langle a|\otimes|a+b+q\rangle\langle a+b|\\&=\sum_{a,b\in\mathbb{Z}_d}\omega^{p(b-a)}\,|a+q\rangle\langle a|\otimes|b+q\rangle\langle b|=X^qZ^{-p}\otimes X^qZ^p\,.\end{aligned}$$

Hence

$$\begin{aligned}CNOT\,w(\vec{a})\,CNOT^*&=\zeta^{-p_1q_1+p_2q_2}(Z^{p_1}\otimes X^{q_2})\,(X^{q_1}Z^{-p_2}\otimes X^{q_1}Z^{p_2})\\&=\zeta^{-p_1q_1+p_2q_2}\zeta^{2q_1p_2-(q_1+q_2)p_2}Z^{p_1-p_2}X^{q_1}\otimes Z^{p_2}X^{q_1+q_2}\\&=\zeta^{p_2(q_1+q_2)}w(p_1-p_2,q_1)\otimes w(p_2,q_1+q_2)\,.\end{aligned}$$

Therefore

$$\partial_{\vec{a}}\,CNOT=\zeta^{p_2(q_2-q_1)}Z^{p_2}\otimes X^{-q_1}\ , \tag{B.22}$$

which follows from

$$\begin{aligned}\partial_{\vec{a}}\,CNOT=w(\vec{a})\,CNOT\,w(\vec{a})^*\,CNOT^*&=\zeta^{p_2(q_1+q_2)}w(p_1,q_1)w(p_2-p_1,-q_1)\otimes w(p_2,q_2)w(-p_2,-q_1-q_2)\\&=\zeta^{p_2(q_1+q_2)+(-p_1q_1-q_1(p_2-p_1))-p_2(q_1+q_2)+q_2p_2}\,w(p_2,0)\otimes w(0,-q_1)\\&=\zeta^{p_2(q_2-q_1)}w(p_2,0)\otimes w(0,-q_1)=\zeta^{p_2(q_2-q_1)}Z^{p_2}\otimes X^{-q_1}\,.\end{aligned} \tag{B.23}$$

Thus

$$||\partial_{\vec{a}}\,CNOT||_{Q^1}=\left|\frac{1}{d^2}\mathrm{Tr}\{\partial_{\vec{a}}\,CNOT\}\right|=\delta_{p_2,0}\delta_{q_1,0}\,. \tag{B.24}$$

It then follows from Proposition 2 in the main text that

$$||CNOT||^4_{Q^2}=\mathbb{E}_{\vec{a}}\,||\partial_{\vec{a}}\,CNOT||^2_{Q_1}=\frac{1}{d^2}\ ,\quad \text{and}\quad ||CNOT||_{Q^2}=\frac{1}{d^{1/2}}\,. \tag{B.25}$$

The case $k \geqslant 3$: For $k = 3$, we use the fact that in Eq. (B.23) we show that $\partial_{\vec{a}}\, CNOT$ is a Weyl operator, up to a phase. Thus $||\partial_{\vec{a}}\, CNOT||_{Q^2} = 1$. Thus using Proposition 2,

$$||CNOT||_{Q^3}^{8} = \mathbb{E}_{\vec{a}}\, ||\partial_{\vec{a}}\, CNOT||_{Q^2}^{2} = 1 \; .$$

Then $||CNOT||_{Q^k} = 1$ also for $k \geqslant 4$.

The qubit case: For $d = 2$ case, i.e., 1-qubit system, the CNOT gate can be written in the computational basis as the following matrix,

$$CNOT = \begin{bmatrix} 1 & 0 & 0 & 0 \\ 0 & 1 & 0 & 0 \\ 0 & 0 & 0 & 1 \\ 0 & 0 & 1 & 0 \end{bmatrix} . \qquad \text{[B.26]}$$

The corresponding quantum uniformity norms are

$$||CNOT||_{Q^1} = \frac{1}{2}, \quad ||CNOT||_{Q^2} = \frac{1}{\sqrt{2}}, \quad ||CNOT||_{Q^k} = 1, \quad \forall k \geqslant 3. \qquad \text{[B.27]}$$

Example 4 (The one-qubit T gate). A single-qubit gate T gate in $\mathcal{C}^3$ plays important role in the universal quantum computation, as Clifford unitary + T gate can generate any unitary operator. The T gate is a fourth root of Z and has the matrix form

$$T = \begin{bmatrix} 1 & 0 \\ 0 & e^{i\pi/4} \end{bmatrix} . \qquad \text{[B.28]}$$

The quantum uniformity norms of T are

$$||T||_{Q^1} = \frac{\sqrt{2+\sqrt{2}}}{2}, \quad ||T||_{Q^2} = \left(\frac{3}{4}\right)^{1/4}, \quad ||T||_{Q^3} = \left(\frac{3}{4}\right)^{1/8}, \quad ||T||_{Q^k} = 1, \forall k \geqslant 4. \qquad \text{[B.29]}$$

The case $k = 1$:

$$||T||_{Q^1} = \frac{1}{2}|\mathrm{Tr}\,\{T\}\,| = \frac{|1+e^{i\pi/4}|}{2} = \frac{\sqrt{2+\sqrt{2}}}{2} = .92387953....$$

The case $k = 2$: Since T commutes with Z,

$$Z^p X^q T X^{-q} Z^{-p} = \begin{cases} \begin{pmatrix} 1 & 0 \\ 0 & e^{i\pi/4} \end{pmatrix} & \text{for } q = 0 \\ \begin{pmatrix} e^{i\pi/4} & 0 \\ 0 & 1 \end{pmatrix} & \text{for } q = 1 \end{cases} , \quad \text{and} \quad \partial_{p,q}\, T = w(p,q) T w(p,q)^* T^* = \begin{cases} \begin{pmatrix} 1 & 0 \\ 0 & 1 \end{pmatrix} & \text{for } q = 0 \\ \begin{pmatrix} e^{i\pi/4} & 0 \\ 0 & e^{-i\pi/4} \end{pmatrix} & \text{for } q = 1 \end{cases} .$$

Thus

$$\left|\frac{1}{2}\mathrm{Tr}\,\{\partial_a\, T\}\right| = \begin{cases} 1 & \text{for } q = 0 \\ \frac{1}{\sqrt{2}} & \text{for } q = 1 \end{cases} . \qquad \text{[B.30]}$$

As a consequence, we can use Proposition 2 to show that

$$||T||_{Q^2}^{4} = \mathbb{E}_a\, ||\partial_a\, T||_{Q^1}^{2} = \mathbb{E}_a \left|\frac{1}{2}\mathrm{Tr}\,\{\partial_a\, T\}\right|^2 = \frac{1}{2}\left(1 + \frac{1}{2}\right) = \frac{3}{4}\;, \quad \text{so} \quad ||T||_{Q^2} = \left(\frac{3}{4}\right)^{1/4} = .93060486....$$

The case $k = 3$: By Proposition 2, we have

$$||T||_{Q^3}^{8} = \mathbb{E}_b\, ||\partial_b\, T||_{Q^2}^{2} = \mathbb{E}_{b,a}\, ||\partial_a \partial_b\, T||_{Q^1}^{2} \; . \qquad \text{[B.31]}$$

Note that

$$\partial_a \partial_b\, T = \begin{cases} \begin{pmatrix} 1 & 0 \\ 0 & 1 \end{pmatrix} , & \text{if } (a,b) = (0,0), (0,1), \text{ or } (1,0) \\ \begin{pmatrix} -i & 0 \\ 0 & i \end{pmatrix} , & \text{if } (a,b) = (1,1) \end{cases} . \qquad \text{[B.32]}$$

Inerting this into Eq. (B.31) shows that

$$||T||_{Q^3}^{8} = \frac{3}{4}\;, \quad \text{and} \quad ||T||_{Q^3} = \left(\frac{3}{4}\right)^{1/8} = .96467863.... \qquad \text{[B.33]}$$

The case $k \geqslant 4$: In this case $\partial_a \partial_b \partial_c T = \pm I$ for all cases of (a, b, c). Thus

$$||T||_{Q^4} = 1 \ , \qquad [B.34]$$

and the same holds for $||T||_{Q^k}$ for $k > 4$.

Example 5 (The CCZ (or Toffoli) gate). The three-qudit gate $CCZ \in \mathcal{C}^3$ (or control-control Z gate) is defined as

$$CCZ = \sum_{x,y,z=1}^{d-1} \omega^{xyz} |x\rangle\langle x| \otimes |y\rangle\langle y| \otimes |z\rangle\langle z| \ , \quad \text{where} \quad \omega = e^{2\pi i/d} \ . \qquad [B.35]$$

The CCZ gate plays an important role in the realization of universal quantum computation. The corresponding quantum uniformity norms are

$$||CCZ||_{Q^1} = \frac{2d-1}{d^2}, \quad ||CCZ||_{Q^2} = \left(\frac{d^3 + (d-1)^3 + d[d^3 - (d-1)^3 - 1]}{d^6} \right)^{1/4}, \qquad [B.36]$$

$$||CCZ||_{Q^3} = \left(\frac{d^3 + d^2 - 1}{d^5} \right)^{1/8}, \quad \text{and} \quad ||CCZ||_{Q^k} = 1, \ \ \forall k \geqslant 4 \ . \qquad [B.37]$$

The case $k = 1$: One can establish this by using Proposition 2,

$$||CCZ||_{Q^1} = \frac{1}{d^3} |\mathrm{Tr}\{CCZ\}| = \frac{1}{d^3} \left| \sum_{x,y,z \in \mathbb{Z}_d} \omega_d^{xyz} \right| = \frac{1}{d^2} \left| \sum_{x,y \in \mathbb{Z}_d} \delta_{xy,0} \right| = \frac{(2d-1)}{d^2} \ .$$

The case $k = 2$: Since CCZ is diagonal in the computational basis, it commutes with Z_j, for $j = 1, 2, 3$. Thus using Proposition 2, and with $\vec{a} = ((p_1, q_1), (p_2, q_2), (p_3, q_3)) \in \mathbb{Z}_d^6$,

$$\begin{aligned} ||CCZ||_{Q^2}^4 =& \mathbb{E}_{\vec{a}} \, ||\partial_{\vec{a}} CCZ||_{Q_1}^2 = \mathbb{E}_{\vec{a}} \, ||w(\vec{a}) \, CCZ \, w(\vec{a})^* \, CCZ^*||_{Q_1}^2 = \mathbb{E}_{\vec{a}} \frac{1}{d^6} \left| \mathrm{Tr} \left\{ w(\vec{a}) \, CCZ \, w(\vec{a})^* \, CCZ^* \right\} \right|^2 \\ =& \mathbb{E}_{\vec{a}} \frac{1}{d^6} \left| \mathrm{Tr} \left\{ (X^{q_1} \otimes X^{q_2} \otimes X^{q_3}) \, CCZ \, (X^{-q_1} \otimes X^{-q_2} \otimes X^{-q_3}) \, CCZ^* \right\} \right|^2 \ . \end{aligned}$$

Evaluating the trace in the computational basis $|k_1\rangle \otimes |k_2\rangle \otimes |k_3\rangle$, we obtain

$$\begin{aligned} ||CCZ||_{Q^2}^4 =& \mathbb{E}_{a,b,c \in \mathbb{Z}_d} \frac{1}{d^6} \left| \sum_{x,y,z} \omega_d^{xyz - (x+a)(y+b)(z+c)} \right|^2 \\ =& \frac{d^3 + (d-1)^3 + d[d^3 - (d-1)^3 - 1]}{d^6}. \end{aligned}$$

And

$$||CCZ||_{Q^3}^8 = \frac{d^3 + 3(d-1)d + 3(d-1)^2 d + (d-1)^3}{d^6} \qquad [B.38]$$

Let us consider a 3-qubit CCZ gate, which has the following form in the computational basis

$$CCZ = \begin{bmatrix} 1 & 0 & 0 & 0 & 0 & 0 & 0 & 0 \\ 0 & 1 & 0 & 0 & 0 & 0 & 0 & 0 \\ 0 & 0 & 1 & 0 & 0 & 0 & 0 & 0 \\ 0 & 0 & 0 & 1 & 0 & 0 & 0 & 0 \\ 0 & 0 & 0 & 0 & 1 & 0 & 0 & 0 \\ 0 & 0 & 0 & 0 & 0 & 1 & 0 & 0 \\ 0 & 0 & 0 & 0 & 0 & 0 & 1 & 0 \\ 0 & 0 & 0 & 0 & 0 & 0 & 0 & -1 \end{bmatrix} . \qquad [B.39]$$

Then we calculate the quantum uniformity norm, and find that

$$||CCZ||_{Q^1} = \frac{3}{4}, \quad ||CCZ||_{Q^2} = \left(\frac{11}{32} \right)^{1/4}, \qquad [B.40]$$

$$||CCZ||_{Q^3} = \left(\frac{11}{32} \right)^{1/8}, \quad ||CCZ||_{Q^k} = 1, \quad \forall k \geqslant 4 \qquad [B.41]$$
